# Meta-analysis on how manure application changes soil organic carbon storage

**DOI:** 10.1038/s41598-021-82739-7

**Published:** 2021-03-09

**Authors:** Arthur Gross, Bruno Glaser

**Affiliations:** Institute of Agricultural and Nutritional Sciences, Soil Biogeochemistry, Martin Luther University Halle-Wittenberg, von-Seckendorff-Platz 3, 06120 Halle/Saale, Germany

**Keywords:** Biogeochemistry, Carbon cycle

## Abstract

Manure application to agricultural soils is widely considered as a source of nutrients and a method of maintaining levels of soil organic carbon (SOC) to mitigate climate change. At present, it is still unclear which factors are responsible for the SOC stock dynamics. Therefore, we analyzed the relationship between SOC stock changes and site characteristics, soil properties, experiment characteristics and manure characteristics. Overall, we included 101 studies with a total of 592 treatments. On average, the application of manure on agricultural soils increased SOC stocks by 35.4%, corresponding to 10.7 Mg ha^−1^. Manure applications in conventional tillage systems led to higher SOC stocks (+ 2.2 Mg ha^−1^) than applications under reduced tillage. Soil organic carbon increase upon manure application was higher in soils under non-tropical climate conditions (+ 2.7 Mg ha^−1^) compared to soils under sub-tropical climate. Larger SOC increases after manure application were achieved in intermediate and shallow topsoils (in 0–15 cm by 9.5 Mg ha^−1^ and in 16–20 cm by 13.6 Mg ha^−1^), but SOC stocks were also increased in deeper soils (> 20 cm 4.6 Mg ha^−1^), regardless of the tillage intensity. The highest relative SOC increase (+ 48%) was achieved if the initial SOC was below 1% but the absolute SOC increased with increasing initial SOC. Clay soils showed higher SOC increase rates compared to sandy soils (+ 3.1 Mg ha^−1^). Acidic soils showed comparable relative effects but a higher stock difference than neutral (+ 5.1 Mg ha^−1^) and alkaline soils (+ 5.1 Mg ha^−1^). The application of farmyard-, cattle- and pig manure showed the highest SOC increases (50%, 32% and 41%, respectively), while green manure and straw showed only minor effects. If manure applications were combined with additional mineral fertilizer, the SOC increases were higher (+ 1.7 Mg ha^−1^) compared to manure alone. Higher applied amounts generally led to higher SOC stocks. However the annually applied amount is only important under conventional tillage, non-tropical climate conditions, and pH-neutral as well as SOC-rich or SOC-depleted soils and if no additional mineral fertilization is applied. Further studies should focus on the SOC dynamics under tropical climate conditions and factors influencing a potential carbon saturation. In both cases, the number of data was too small. For this reason, additional field studies should be conducted primarily in the tropics. On the other hand, long-term field trials should be re-assessed or newly established to specifically investigate potential saturation effects and long-term (> 20 years) fertilizer effects and carbon sequestration.

## Introduction

The continuously rising concentrations of atmospheric greenhouse gases (GHG) due to human and natural emissions are the main drivers of climate change^1^. This necessitates approaches for mitigating GHG emissions. Strengthening renewable energies or mitigating emissions using carbon capture and storage (CCS) or carbon capture and use (CCU), are possible GHG mitigation options^2^. However, since the switch to renewable energy supplies is still limited by political and market barriers, and geological storage technologies such as CCS are associated with certain risks^3^, they have less acceptance^4^. Nonetheless, there remains a need for sustainable and safe carbon removal from the atmosphere.

Soils are an important carbon sink, as they contain more carbon than stored in terrestrial vegetation and the atmosphere combined^5^. Several regional studies showed that there is still potential to store even more carbon in soils, if certain management practices are applied^6,7^. This process of storing organic carbon in soils, better known as carbon sequestration, describes how organic carbon is put into soils and converted into a stabilized form in the long-term (> 100 years). Besides its beneficial climatic effects, higher soil organic carbon (SOC) content promotes several important soil functions, such as nutrient transformation and supply, soil–water balance control or buffering of pollutants^8^. In short, soil organic matter (SOM) is important for adapting to climate change as well as mitigating it.

Theoretically, there are many ways of increasing the SOC pool^9^. However, most of their practical potential is limited. For instance, while no tillage did not significantly increase SOC stocks^10^, cover crops increased SOC stock by 9–10%, based on a review of global meta-analysis data^11^. Another option is to use different organic materials with high carbon content as soil amendments. Manure is a collective term for excrements of different animal species, urine, plant materials and straw but also livestock feed residues and human household waste. Manure nitrogen production increased from 21.4 Tg N year^−1^ in 1860 to 131.0 Tg N year^−1^ in 2014 with an annual increasing trend of 0.7 Tg N year^−1^ ^12^. Cattle dominated the manure nitrogen production and contributed ∼ 44% of the total manure nitrogen production in 2014, followed by goats, sheep, swine, and chicken^12^. The manure nitrogen application to cropland accounts for less than one-fifth of the total manure nitrogen production^12^. Manure might also increase carbon in soils as these materials have high carbon content. However, organic matter in manure might also be easily degraded due to its high nitrogen content or its low carbon-to-nitrogen ratio. Many individual studies measured the impact of manure application on SOC stocks, with few studies showing increases in SOC stocks, but also studies showing only small or even negative impacts. Due to this wide variation in results, there is a need for studies clarifying factors that control the magnitude of change in SOC stocks due to manure application. Up to now, only two quantitative reviews have tried to find global relationships between the magnitude in SOC stock changes and different explanatory factors. Han et al*.*^13^ focused on combined treatments of manure and mineral fertilizer and Maillard and Angers^14^ included studies with mineral fertilizer as reference to manure treatments. Furthermore, Maillard and Angers^14^ only considered articles published up to 2011.

Due to this current lack of clear evidence and statistically significant relationships between SOC stock changes upon manure application and global explanatory factors, we conducted a meta-analysis. The aim of this study was to calculate the response ratio of carbon stocks to manure application and the SOC stock difference under consideration from data available from peer-reviewed studies (ISI Web of Science). Furthermore, our target was to identify clear evidence of influencing factors. For this purpose, we grouped and analyzed the results according to the following criteria: site characteristics (climate zone), soil properties (initial SOC content, pH value, soil texture), experiment characteristics (tillage intensity, experiment duration, sampling depth) and manure characteristics (manure type, added manure amount, additional mineral NPK fertilizer). In addition to the analysis within individual categories, we also examined intercategorical effects to investigate possible interactions between the investigated factors.

## Material and methods

### Data sources, collection and categorization

In order to analyze SOC stock changes following manure application, a meta-analysis was conducted. Within this framework, we performed a systematic literature review using “ISI Web of Science (Core Database)”. The search term was “(Soil organic matter OR C Sequestration) AND Manure”. Studies were included if they were performed under field conditions and if the effect and control size was expressed as content of total organic carbon (TOC) or quantified as SOC or TOC stocks. If SOM rather than SOC information were given in a study, we calculated SOC as SOM multiplied by 0.58. All treatments with a duration of ≤ 3 years were removed to exclude short-term effects and the influence of the cultivated crops. Overall, 101 studies with a total of 592 treatments were included.

Besides information on SOC content, we also extracted information on soil properties (initial SOC content, texture, bulk density, soil pH class), experiment characteristics (tillage intensity, duration, sampling depth), manure characteristics (type, added amount, additional mineral fertilizer use) and site characteristics (longitude, latitude, altitude, climate zone). To limit the variety of different soil texture classes, we decided to group them into their respective dominant particle size class (sand, silt or clay). Exceptions are the middle classes "clay loam and loam". These have been added to "loam". If data were only presented in figures, WebPlotDigitizer Version 4.2 was used for the extraction of data. In order to analyze the total amount of manure added, annual amounts were accumulated.

If no information on SOC stocks was provided, we quantified them using the following Eq. (1)^15^,

1 $$SOC\, stock=SOC\times Bulk \,density\times Depth\times 0.1$$

where SOC stock is expressed as Mg ha^−1^, bulk density as g cm^−3^, depth as cm and SOC as g kg^−1^. In a few studies, no soil bulk density was given. In these cases, we used different pedotransfer functions. If studies included information on the initial SOC, silt and clay content, we used the pedotransfer function given in Men et al*.*^16^ (Eq. 2). If studies included information on the initial SOC and the clay content, we used an equation given in Bernoux et al*.*^17^ (Eq. 3). If studies only provided information on initial SOC, we used a pedotransfer function given in Manrique and Jones^18^ (Eq. 4).

2 $$Bulk \,density = 1.386 - 0.078 \times SOC + 0.001 \times Silt + 0.001 \times Clay$$

3 $$Bulk\, density = 1.398 - 0.0047 \times Clay - 0.042 \times SOC$$

4 $$Bulk \,density = 1.660 - 0.318 \times {SOC}^{0.5}$$

where bulk density is expressed as g cm^−3^ and the SOC, silt and clay content as %. To better understand the factors influencing SOC stock changes, we grouped the study results as follows: tillage intensity type, climate zone, initial SOC, soil texture, sampling depth, soil pH class, added annual manure amount, cumulative manure amount, manure type, additional mineral fertilizer and experiment duration.

### Data analysis

To estimate the effects of manure applications on SOC stock changes, we used two different indices. We calculated the response ratio (R), which is the mean of the manure treatment divided by the mean of the control group (all the same but without manure application) and we calculated the SOC stock mean difference (∆SOC). To measure experimental effect sizes, R and ∆SOC are both very common and wide-spread in meta-analyses^13,14,19–21^. It is essential to calculate both indices as R only gives information on relative changes whereas ∆SOC considers the absolute impact. The consideration of only one of those indices can be misleading. Two similar absolute SOC changes can be the result of either a low or a high relative SOC change, depending on the initial SOC content.

R was calculated using the following equation:

5 $$R=\left(\frac{{X}_{E}}{{X}_{C}}\right)-1$$

where X_E_ is the mean SOC stock with manure application and X_C_ is the mean SOC stock without application of manure (control group) for each treatment. In order to better interpret the result, 1 was subtracted from each R value.

More precise meta-analysis are using a weighting according to the number of repetitions, the standard deviation or the standard error. Considering the fact that only a few of the analyzed studies provided sufficient information on statistical measures and replicates, we decided to use un-weighted meta-analysis, to include as many treatments as possible. “Un-weighted” meta-analysis is a commonly used approach, which gives all included studies the same weight, e.g. a weight of 1^13,22–24^. SOC stock differences were calculated by using Eq. (6):

6 $$\Delta SOC={X}_{E}-{X}_{C}$$

where X_E_ represents the mean SOC stock in Mg ha^−1^ of the experimental group and X_C_ the mean SOC stock in Mg ha^−1^ of the control group.

For reasons of better interpretation, 95% confidence intervals (CI) were calculated as follows:

7 $$CI \,upper=R or \Delta SOC\frac{+1.96*\sigma }{\sqrt{n}}$$

8 $$CI\, lower=R or \Delta SOC\frac{-1.96*\sigma }{\sqrt{n}}$$

with the mean response ratio R or the SOC stock mean difference ∆SOC in Mg ha^−1^, 1.96 the confidence coefficient, *σ* the standard deviation and n the number of individual treatments.

All of these statistical measures are presented as forest plots. Visualisation was conducted with R Version 3.5.2^25^. The overall grand mean of all individual treatments is presented in the first row. The grey solid line represents an R, or a mean difference equal to 0, thus no effect. An effect size larger than 0 indicates a positive effect (i.e. an increase of SOC upon manure application), and lower than 0 a negative effect (i.e. a decrease of SOC upon manure application). Each effect size is presented as the range between the upper and lower 95% confidence interval. The line inside of both confidence intervals represents the range of the effect size. The range between both confidence intervals of the grand mean is shown by the extent of the rectangle. If the effect size range crosses the “zero-effect-line”, the result can be interpreted as statistically insignificant. The mean effect sizes of each group were considered to be significantly different at p < 0.05 from each other if the 95% confidence intervals were not-overlapping. The number after the name of the examined category represents the number of included treatments.

In the inter-categorical evaluation, all influencing factors were compared with each other. Due to the resulting large number of data, we decided to examine only those intermediate category treatments that occurred at n ≥ 10. Furthermore, we eliminated all treatments in the intercategorical evaluation, which applied combinations of manure types due to too many different combinations and, therefore, too few repetitions per manure treatment class.

To analyze the connection of ∆SOC and added manure amounts under the influence of various factors, a linear regression analysis was conducted using R Version 3.5.2^25^. We calculated the coefficient of determination R^2^ and the statistical connection was determined by using the Pearson correlation coefficient R. Normal distribution was checked using the Shapiro–Wilk test.

## Results and discussion

### General effect

Overall, 101 studies with a total of 592 treatments were analyzed in this study (Supplementary Dataset 1). All of them were conducted under field conditions. No laboratory experiments were included. Locations in North America (n = 8), South America (n = 2), Sub-Saharan Africa (n = 5), Europe (n = 11), West Africa (n = 2), South Asia (n = 20) and East Asia (n = 53) were included. The results of all subcategories, including their standard deviation can be found in the Supplementary Dataset 2. The results of the intercategorical grouping is located in the Supplementary Dataset 3 and their corresponding forest plots can be found in the Supplementary Material.

As expected, the results obtained from 592 pairwise comparisons showed a significant increase of SOC stocks of 35% (95% CI 32–39%) and a ∆SOC of 10.7 Mg ha^−1^ (95% CI 9.8–11.6 Mg ha^−1^) on average, after manure was applied despite high variation among different groups. This positive effect can mainly be explained by the fact that manure applications are direct inputs of carbon into soil and a source of nutrients (especially nitrogen), which results in an increased net primary production of plants and increased yields^26–28^. Increasing plant primary production leads to an increase of crop residue inputs and rhizodeposition, which both enhance SOC sequestration^29^.

### Tillage intensity effect

**Figure 1:**
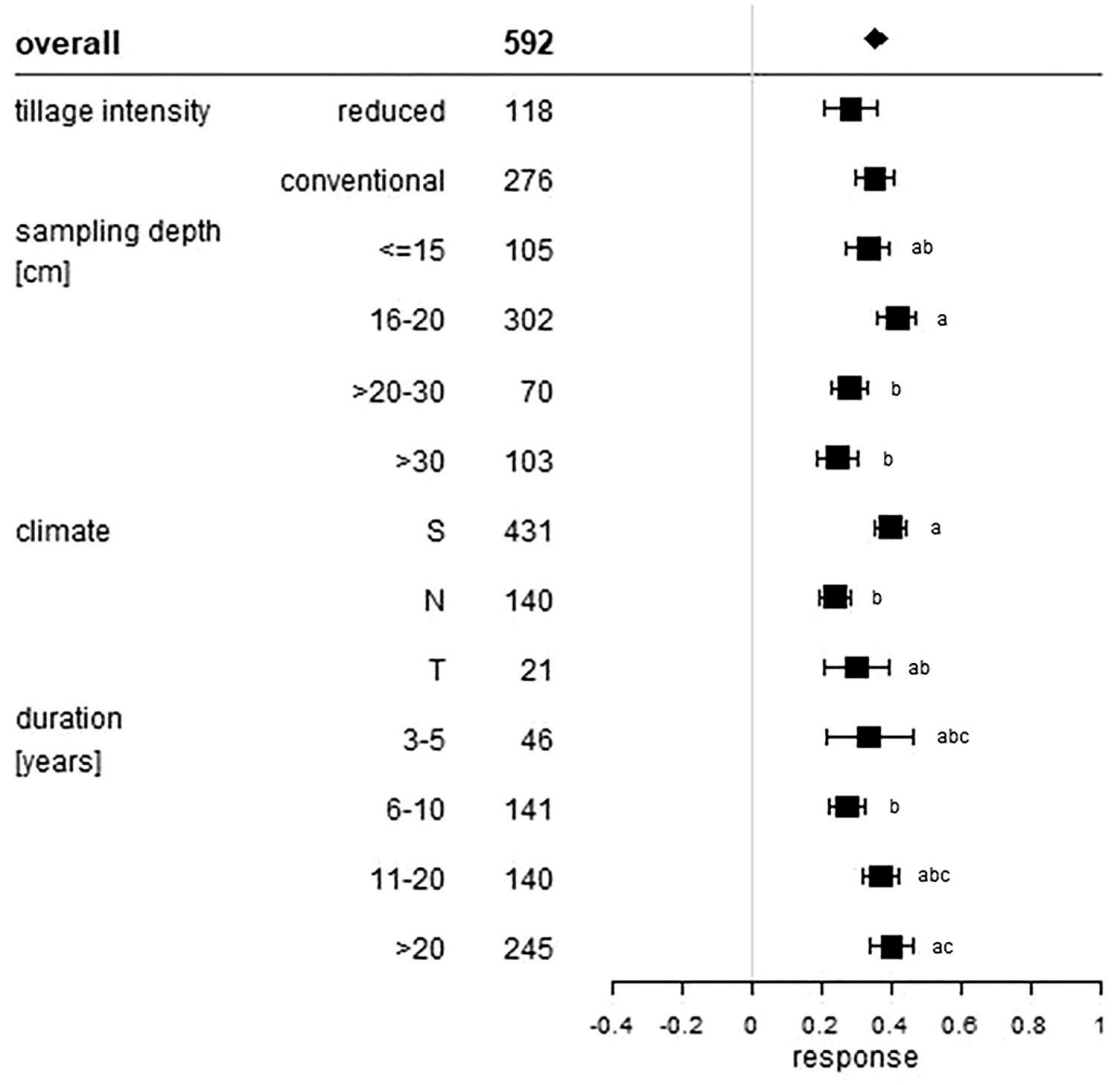
Relative response of manure applications on soil organic carbon stocks influenced by tillage intensity, sampling depth (cm), climate and duration (years) of the considered treatments. The overall grand mean of all individual treatments is presented in the first row followed by the considered subcategories below. Each response ratio is presented as the range between the upper and lower 95% confidence intervals. Points within the range represent the mean response ratio. The range between both confidence intervals of the grand mean is shown by the extent of the rectangle. The number in each treatment row represents the number of pairwise comparisons on which the statistic is based. The grey line was drawn at response ratio = 0. Different letters in each subcategory indicate statistical significant differences.

**Figure 2:**
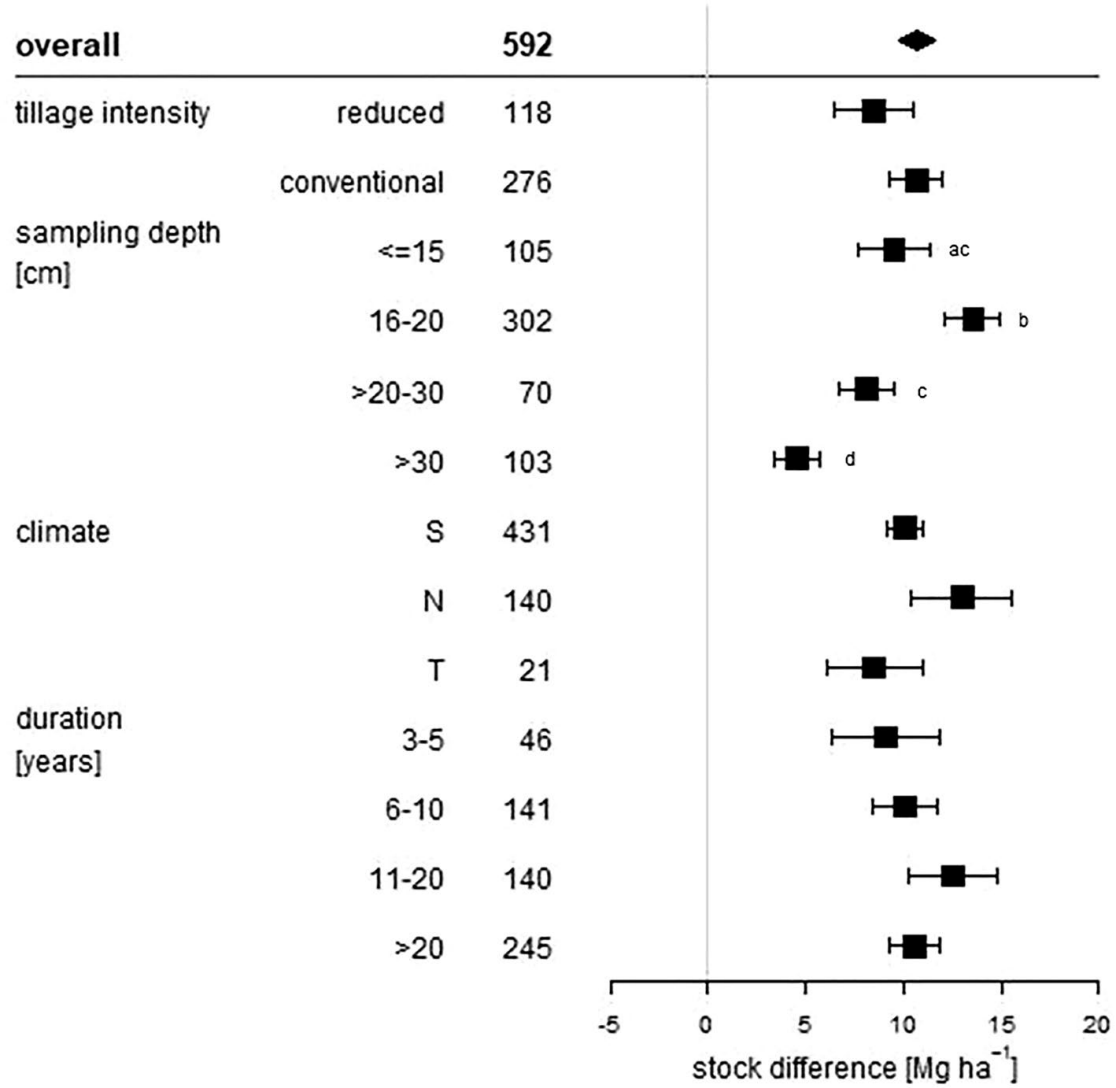
Absolute response (Mg ha^−1^) of manure applications on soil organic carbon stocks influenced by tillage intensity, sampling depth (cm), climate and duration (years) of the considered treatments. The overall grand mean of all individual treatments is presented in the first row followed by the considered subcategories below. Each response is presented as range between the upper and lower 95% confidence intervals. Points within the range represent the mean response. The range between both 95% confidence intervals of the grand mean is shown by the extent of the rectangle. The number in each treatment row represents the number of pairwise comparisons on which the statistic is based. The grey line was drawn at stock difference = 0 Mg ha^−1^. Different letters in each subcategory indicate statistical significant differences.

Out of 592 treatments that we analyzed, 394 treatments provided information on tillage intensity. 276 treatments were conducted on soils under conventional tillage and 118 treatments on reduced tillage soils. The relative SOC change of the tillage intensity group is presented in Fig. 1. Both treatments showed positive magnitudes with a mean increase of SOC stocks of 35% for conventional tillage and 28% for reduced tillage systems. ∆SOC was 10.7 Mg ha^−1^ for conventional tillage and 8.5 Mg ha^−1^ in reduced tillage systems (Fig. 2). It is known that reduced tillage has beneficial effects on soil quality, e.g. physical, biological and chemical properties^30–32^, but the effects of tillage on carbon accumulation are controversially discussed. While many studies showed higher SOC accumulation in reduced tillage systems after manure application^33–35^, Baker et al*.*^36^ argued that SOC accumulation caused by reduced tillage are biased, as most of the studies conducted only involved shallow sampling. Studies which involved deeper sampling often show no positive or insignificant sequestration effects^36^. Our results point to different dynamics. The intercategorical evaluation of tillage intensity and sampling depth shows that manure applications even under reduced tillage led to the smallest but still a significant enrichment of SOC in depths > 30 cm (Supplementary Material, Figure S1 and S2). ∆SOC increased by 3.7 Mg ha^−1^ corresponding to an R of 19%. Conventional tillage in depths > 30 cm led to a SOC increase of 23% corresponding to 5.6 Mg ha^−1^. Shallow sampling depths ≤ 15 cm led to a SOC increase of 21% under reduced tillage and a ∆SOC of 7.2 Mg ha^−1^. Under conventional tillage, shallow sampling depths showed a larger SOC increase of 40% and also a larger ∆SOC of 9.0 Mg ha^−1^. Overall, sampling depth-wise both tillage intensities showed the same SOC increase with large relative and absolute SOC increases in shallow soil depths and smaller responses in deeper regions. This seems logical, as manure applications under conventional tillage are usually only ploughed into the soil up to a depth of 20–30 cm, or in the case of reduced tillage only very shallowly or not at all.

Regarding sampling depth, a 12% lower SOC stock response was observed at near-surface sampling depth equal to or less than 15 cm, compared to 16–20 cm soil depth (Fig. 1). If sampling depth was > 20–30 cm, SOC stock increased by 28%. In > 30 cm soil depth, SOC increased by 24%. ∆SOC showed higher results in near-surface regions than in greater soil depth, with 9.5 Mg ha^−1^ in the first 15 cm and 4.6 Mg ha^−1^ in depths > 30 cm (Fig. 2). The largest ∆SOC was achieved in 16 – 20 cm soil depth with 13.6 Mg ha^−1^. The vertical distribution of SOC in agriculturally used soils can differ largely, depending on the applied tillage practice^37^. Where SOC accumulates in the soil surface in reduced- or no tillage systems, ploughing in conventional systems can lead to a shift of SOC in deeper soil regions^38^. However, our results showed no significant differences in ∆SOC in shallow regions of conventional tillage soils compared to reduced tillage soils. Conventionally tilled soils showed a larger relative response, but again this difference was not significant. This result corresponds to findings of a meta-analysis of cover crop induced SOC effects, where also no significant differences between SOC stocks of conventional and reduced tillage soils could be identified^10^.

### Climate effect

Figure 1 presents the relative SOC stock change induced by climatic conditions. The lowest response ratio was observed in non-tropical climates, with an average SOC stock increase of 24%. SOC stock changes in tropical climates had a positive mean value of 30%. The highest positive response, with an average of 40%, was accounted for sub-tropical climates. Tropical climate responses showed a large range and were not significantly different from the other climatic categories due to the low number of only 21 treatments. ∆SOC showed different dynamics. The highest difference of 12.8 Mg ha^−1^ was reached under non-tropical climatic conditions (Fig. 2). Subtropical and tropical conditions led to lower ∆SOC of 10.1 Mg ha^−1^ and 8.5 Mg ha^−1^, respectively. The relative and absolute SOC changes confirms common paradigm. Generally, soils in cool and humid climates have a larger potential to store SOC than soils in dry and warm regions, due to lower decomposition rates and, therefore, higher carbon accumulation^9^. Also Maillard and Angers^14^ showed that the absolute difference in SOC stocks after manure application is lower in tropical and warm regions than in cool regions^14^. The response ratio results can be explained by initial SOC content and stocks in tropical and sub-tropical soils, which are generally lower than in soils of cooler regions^9^. SOC-poor soils have a larger potential to store additional SOC than SOC-rich soils and, therefore, have higher initial SOC accumulation rates^39^. This leads to a larger relative SOC increase in sub-tropical and tropical soils, compared to SOC-richer soils in non-tropical regions. Our results of the intercategorical grouping of the climate categories and initial SOC partly confirm this understanding (Supplementary Material, Figure S3 and S4). Out of 263 treatments under subtropical conditions, which reported initial SOC values, 160 reported an initial SOC content < 1%. These treatments showed a large response ratio of 48% but low ∆SOC results of 10.3 Mg ha^−1^. In turn, most of the treatments under non-tropical conditions which reported SOC, showed an initial SOC content > 2%. These treatments were characterized by a low mean response ratio of 23% but a high SOC stock difference of 28 Mg ha^−1^. Due to low number of samples (n = 23), the error bar is wide and limits a conclusive statement. The analysis under tropical conditions was only possible for intermediate initial SOC contents due to the small number of samples.

### Temporal effect

The relative SOC stock response connected with the durations of the experiments are shown in Fig. 1. Four different durations were analyzed. The relative mean stock increase of treatments with durations between 3 and 5 years was 34%. If experiments had durations between 6 and 10 years, SOC stocks increased by 27%. Between 11 and 20 years, mean response ratio was 36%. For durations higher than 20 years, SOC stocks changed by 40%. The highest stock difference was gained between 11 and 20 years and > 20 years, with a ∆SOC of 12.5 and 10.6 Mg ha^−1^ (Fig. 2). If the duration was between 3 and 5 years, stocks only changed by 9.1 Mg ha^−1^, but showed a high relative gain. This is not surprising, as initial SOC accumulation rates are generally high if the area is feasible and management practices are of good choice^7^. As there is no large difference in the effect sizes (relatively and absolutely) between durations between 3 and 5 years and more than 20 years, SOC stocks do not change systematically with time, if applied manure amounts did not differ interannually. This finding indicates the potential of manure to store carbon in the long term. But, as SOC stocks do not increase with duration, carbon saturation is indicated. The timing of saturation not only depends on soil and input material properties, but also on the initial SOC content. Soils with high initial SOC content reach carbon saturation within a short period of time, whereas soils with low initial SOC need more time^40^. West and Six showed that carbon saturation might occur over a period of 26 years under conventional rotation and 21 years under no till^39^. Moreover, the saturation equilibrium seem to depend on the soil texture, sandy soils being more prone to C saturation^41^. According to Wiesmeier et al*.*, finer textured soils showed a depletion of SOC^42^. Due to the variety of different factors influencing SOC saturation, we further analyzed the intercategorical effect of the initial SOC content, soil texture and tillage intensity on the temporal SOC storage dynamics.

Our findings regarding the influence of initial SOC on carbon saturation supports the common paradigm. Treatments with low initial SOC show large relative responses in all durations and no depletion over time in absolute terms (Supplementary Material, Figure S5). Treatments with intermediate initial SOC showed lower relative responses and a depletion of ∆SOC between durations between 11 and 20 years and durations > 20 years (Supplementary Material, Figure S6).

With regard to the tillage intensity, the experiment duration does not seem to play a major role. All response ratios show wide ranges and quite similar mean values but slight increases regarding long experimental duration > 20 years with 32% under reduced and 40% under conventional tillage (Supplementary Material, Figure S7). The ∆SOC results also do not allow a meaningful conclusion (Supplementary Material, Figure S8). Error bars are too wide and mean values are too similar. However, a slight depletion is indicated under conventional tillage at durations > 20 years. Here, the ∆SOC showed a lower response of 9.3 Mg ha^−1^.

Texture-wise the situation is different. Mean response ratios were high in sandy soils with experimental duration between 11 and 20 years and > 20 years with 56% and 40% respectively (Supplementary Material, Figure S9). However, with respect to SOC stocks, both analyzed durations did not differ largely and showed a low level (Supplementary Material, Figure S10). Fine textured clay soils however, showed both high relative and absolute mean responses in large durations with 74% and 21.3 Mg ha^−1^ in durations between 11 and 20 years. Our results therefore seem to contradict the statement that clay soils show SOC depletions over time. But a more conclusive statement requires more long-term experiments with durations > 20 years.

### Soil properties effect

**Figure 3:**
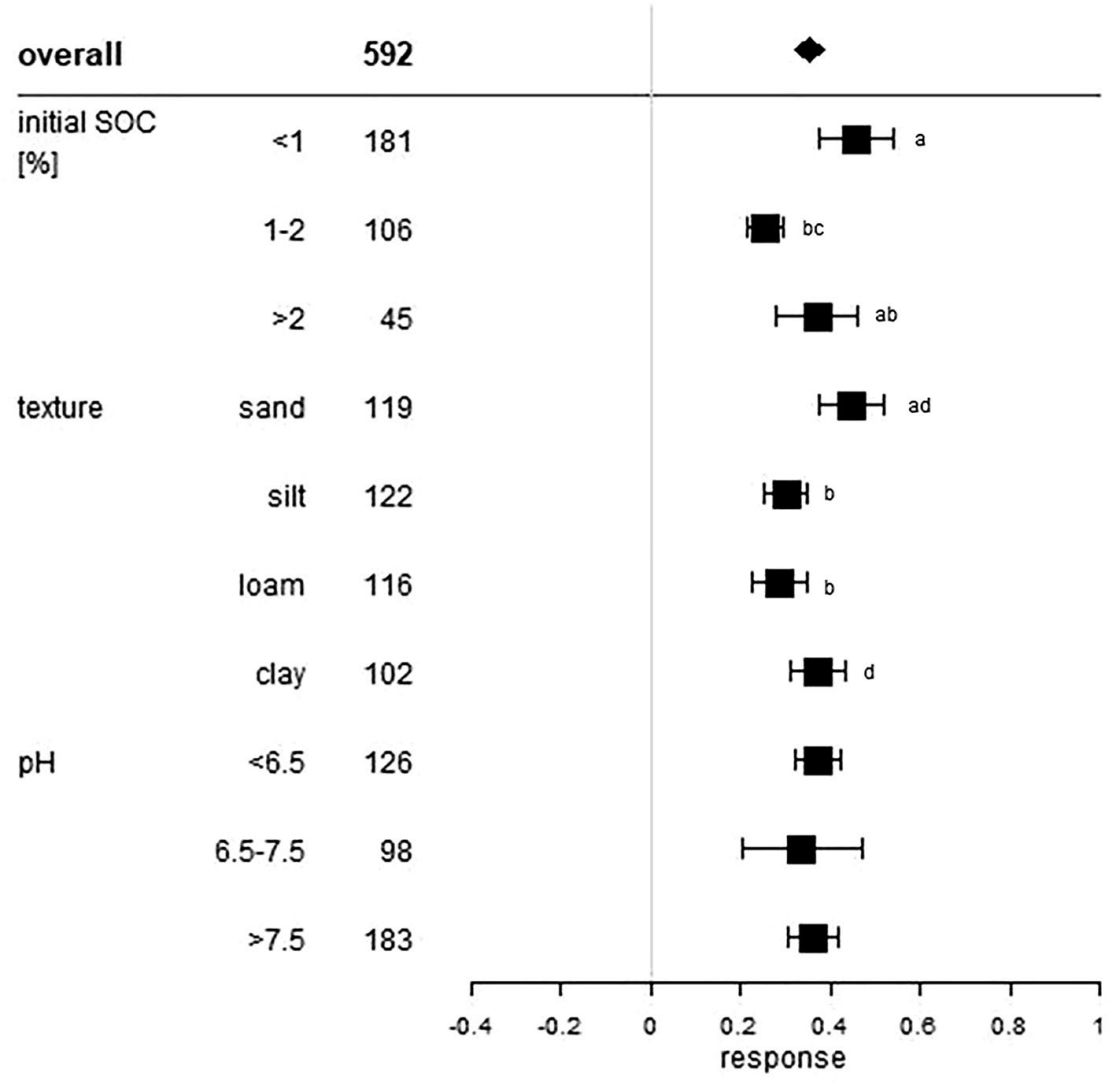
Relative response of manure applications on soil organic carbon stocks influenced by the initial soil organic carbon content (%), soil texture and soil pH value of the considered treatments. The overall grand mean of all individual treatments is presented in the first row followed by the considered subcategories below. Each response ratio is presented as the range between the upper and lower 95% confidence intervals. Points within the range represent the mean response ratio. The range between both 95% confidence intervals of the grand mean is shown by the extent of the rectangle. The number in each treatment row represents the number of pairwise comparisons on which the statistic is based. The grey line was drawn at response ratio = 0. Different letters in each subcategory indicate statistical significant differences.

**Figure 4:**
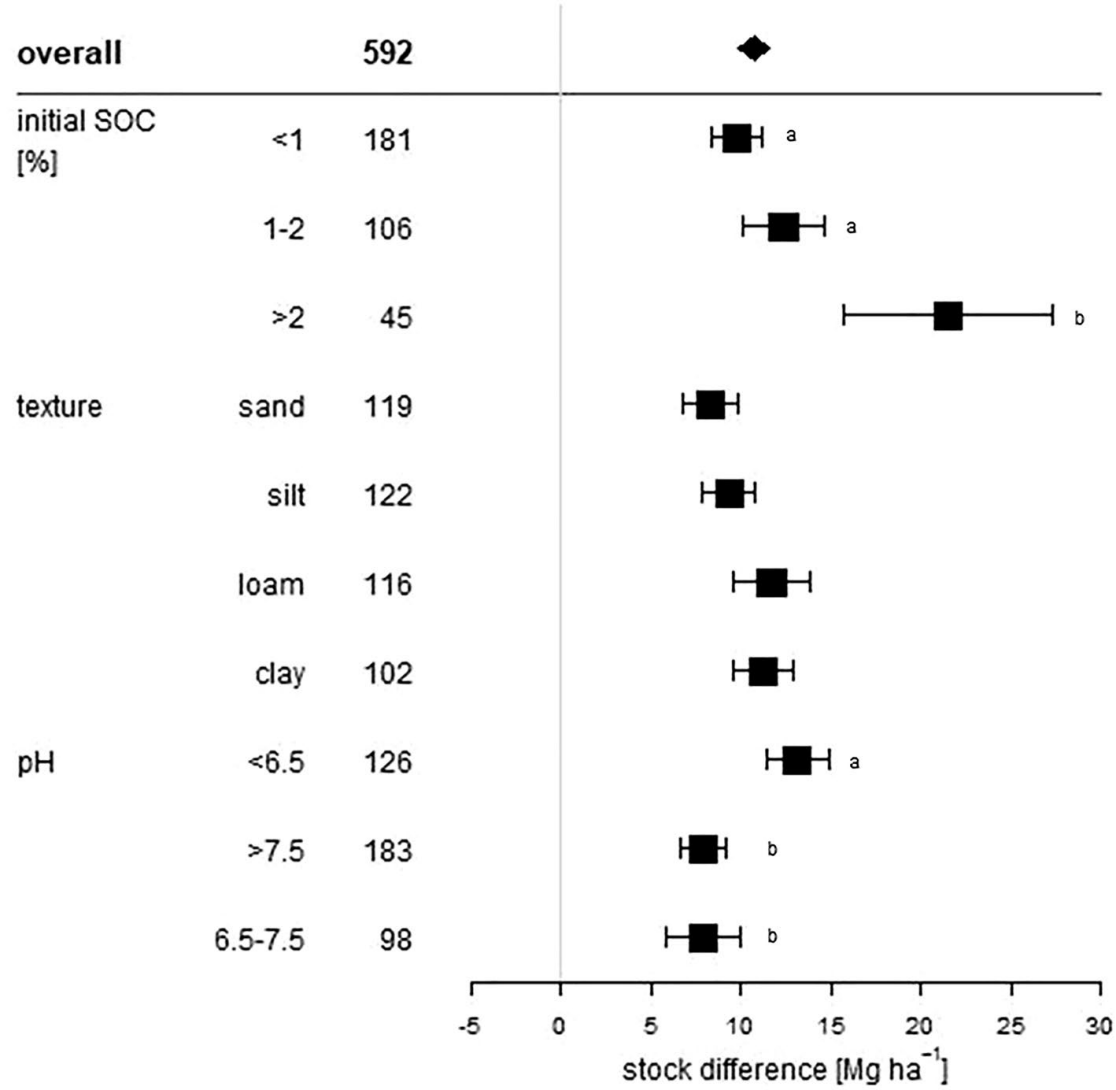
Absolute response (Mg ha^−1^) of manure applications on soil organic carbon stocks influenced by the initial soil organic carbon content (%), the soil texture and the soil pH value of the considered treatments. The overall grand mean of all individual treatments is presented in the first row followed by the considered subcategories below. Each response is presented as the range between the upper and lower 95% confidence intervals. Points within the range represent the mean response. The range between both 95% confidence intervals of the grand mean is shown by the extent of the rectangle. The number in each treatment row represents the number of pairwise comparisons on which the statistic is based. The grey line was drawn at stock difference = 0 Mg ha^−1^. Different letters in each subcategory indicate statistical significant differences.

The influence of initial SOC content on the relative effect size within our analysis is presented in Fig. 3. Treatments with low initial SOC content < 1% (46%) showed higher stock increases than treatments with initial SOC content between 1 and 2%, with stock increases of 25%. Treatments with initial SOC content > 2% resulted in a mean response of 37%. ∆SOC results were highest in treatments with initial SOC > 2% (21.5 Mg ha^−1^) and were lower with decreasing initial SOC content with 12.4 Mg ha^−1^ in treatments with 1 – 2% initial SOC and 9.8 Mg ha^−1^ in treatments with initial SOC < 1% (Fig. 4). This finding is not unexpected as even small relative SOC stock changes in soils with high SOC content are leading to large absolute stock differences. In turn, large relative changes in soils with a low initial SOC are leading to a low absolute difference. Soils with an intermediate initial SOC showed an intermediate absolute stock difference. The relative effect of intermediate initial SOC content was the lowest of all responses but the difference was not significant compared to high initial SOC content.

All soil texture classes had positive significant responses on SOC stocks after manure was applied, but responses differed largely between the texture classes (Fig. 3). There are different processes related to soil carbon stabilization as a function of soil texture. These processes often depend on the soil clay content. Sorption processes of SOC on soil mineral surfaces and SOC incorporation within soil aggregates can both be enhanced by higher content of clay-sized particles, as clay-sized particles have a higher reactive surface area than coarser particles^43^. This supports our findings that texture classes with higher clay content showed significantly increased ∆SOC in loam and clay soils with mean differences of 11.7 and 11.3 Mg ha^−1^ (Fig. 4). Furthermore, clay soils also showed high relative SOC increases (37%), suggesting that soils with small particle sizes are best suited for SOC storage. Due to the lower specific surface area, sandy soils tend to have higher leaching losses of dissolved organic carbon than finer soil material and are usually more aerated, which favors SOC decomposition. Both processes underpin our finding concerning manure application on sandy soils. Results showed a high relative increase of 45% but the lowest of all analyzed absolute SOC responses with 8.2 Mg ha^−1^. The high relative increase seems to be related to low initial SOC values, which in our evaluation often occurred in sandy soils (n = 51) (Supplementary Material, Figure S11 and S12). Out of all initial SOC value classes, only the < 1% class could be evaluated because higher initial values occurred too rarely (n < 10).

Soils with pH < 6.5 showed a mean response of 37% and a mean SOC increase of 13.1 Mg ha^−1^. Neutral soils (6.5–7.5) had a mean SOC increase of 7.9 Mg ha^−1^ corresponding to 25%, while alkaline soils (> 7.5) showed a SOC increase of 7.9 Mg ha^−1^ corresponding to 36% (Figs. 3, 4). The supply of protons to soils, from atmospheric or organic sources, influences several biological and chemical processes e.g. soil microbial activity, which affects decomposition of organic matter and carbon sequestration^44^. Generally, increasing soil pH stimulates microbial activity and decomposition rates of fresh organic matter and, therefore, favors SOC mineralization^45^. However, it is still unknown whether a higher net primary production as a consequence of raising soil pH (e.g. through lime application) and, therefore, higher plant residue and root biomass inputs could possibly offset higher soil respiration and promote carbon sequestration in the long term^46,47^. Furthermore, a higher amount of Ca^2+^ ions could favor formation of mineral-organic complexes in soils with higher pH. Decreasing pH values, in turn, can reduce decomposition rates of SOC^48^. Therefore, acidity could possibly promote SOC accumulation which our results confirmed as manure application showed the highest absolute and relative SOC stock responses in acid soils.

### Fertilizer properties and amount effect

**Figure 5:**
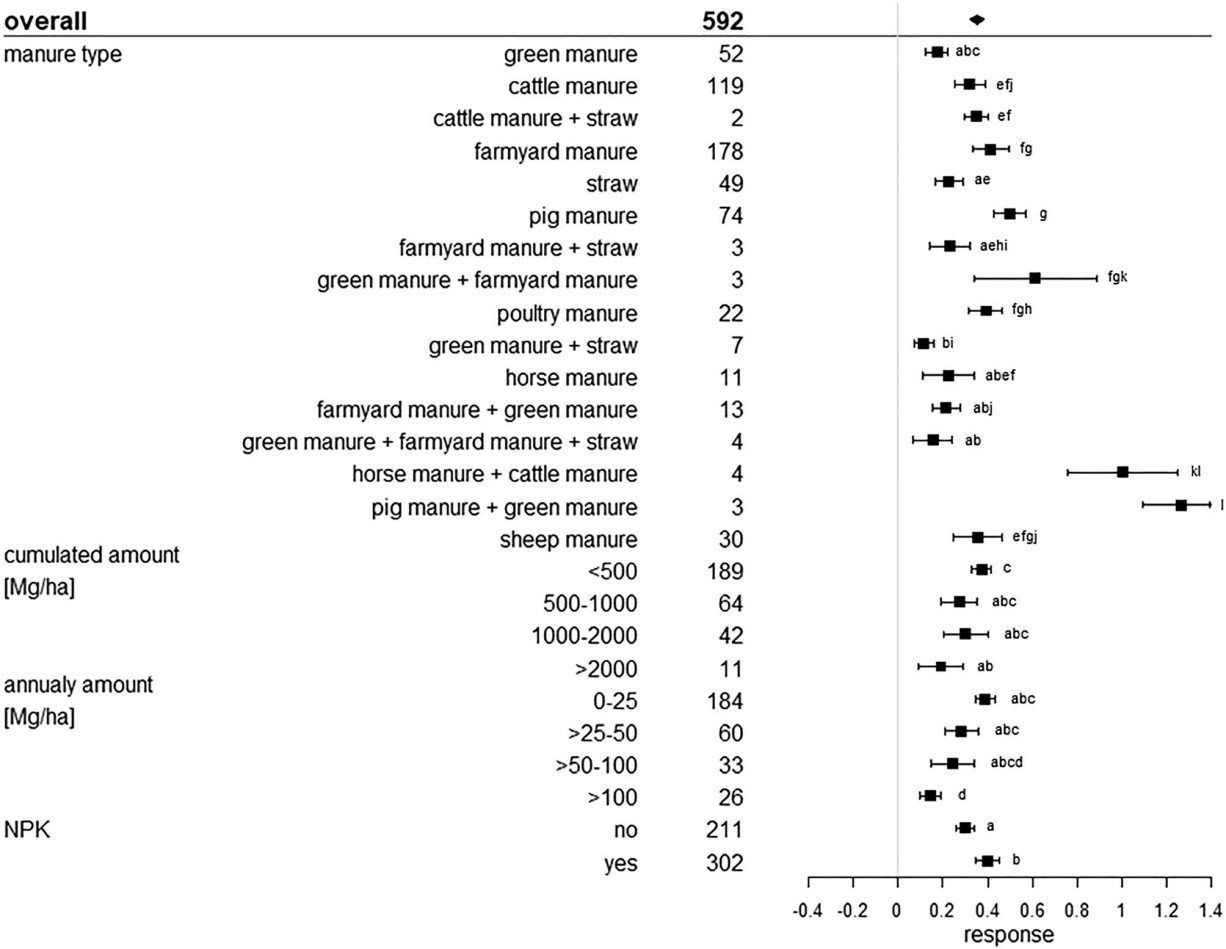
Relative response of manure applications on SOC stocks influenced by manure type, annual manure amount (Mg ha^−1^), the accumulated manure amount (Mg ha^−1^), and additionally added chemical fertilizer (NPK) of the considered treatments. The overall grand mean of all individual treatments is presented in the first row followed by the considered subcategories below. Each response ratio is presented as the range between the upper and lower 95% confidence intervals. Points within the range represent the mean response ratio. The range between both 95% confidence intervals of the grand mean is shown by the extent of the rectangle. The number in each treatment row represents the number of pairwise comparisons on which the statistic is based. The grey line was drawn at response ratio = 0. Different letters in each subcategory indicate statistical significant differences.

**Figure 6:**
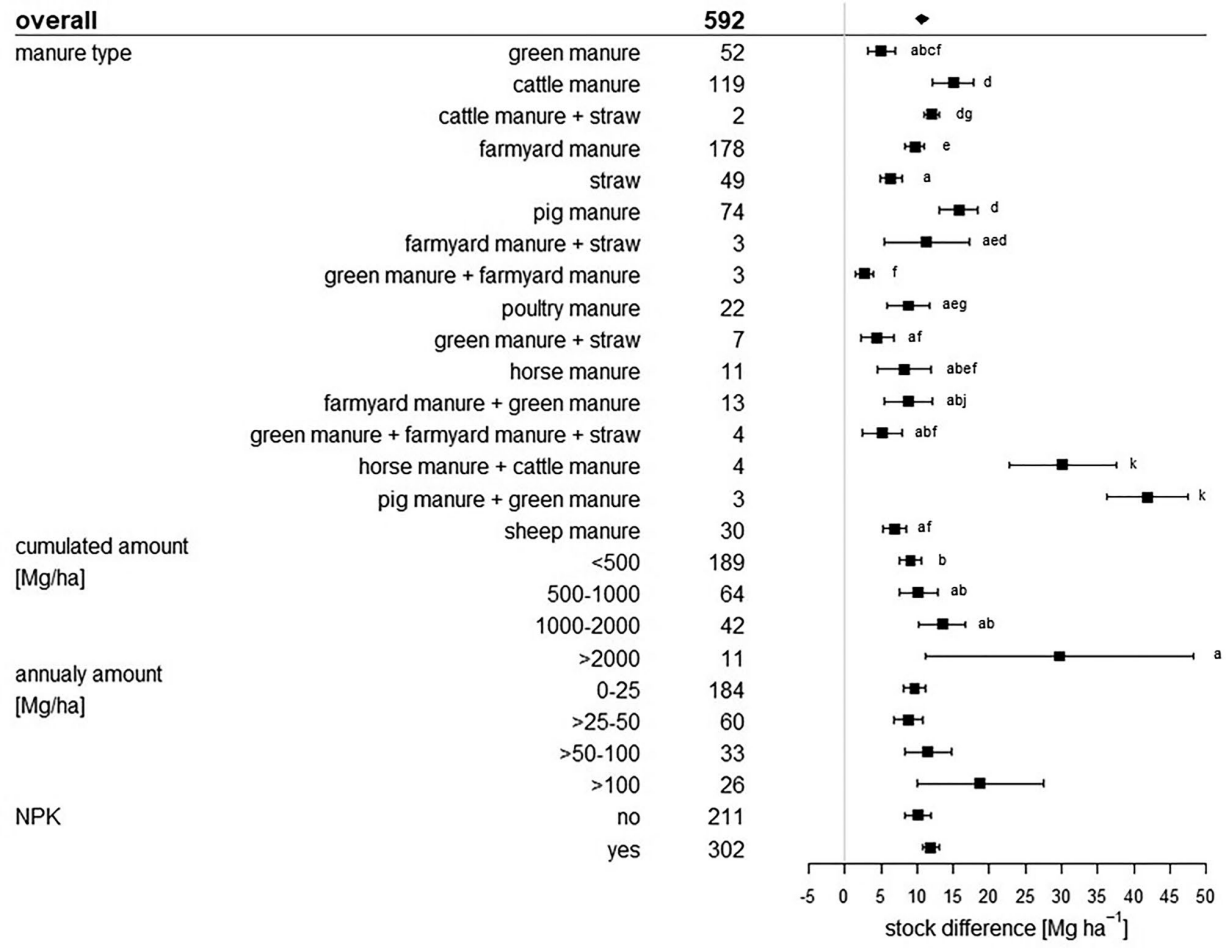
Absolute response (Mg ha^−1^) of manure applications on soil organic carbon stocks influenced by the manure type, the annual manure amount (Mg ha^−1^) the accumulated manure amount (Mg ha^−1^) and additional added chemical fertilizer (NPK) of the considered treatments. The overall grand mean of all individual treatments is presented in the first row followed by the considered subcategories below. Each response is presented as the range between the upper and lower 95% confidence interval. Points within the range represent the mean response. The range between both 95% confidence intervals of the grand mean is shown by the extent of the rectangle. The number in each treatment row represents the number of pairwise comparisons on which the statistic is based. The grey line was drawn at stock difference = 0 Mg ha^−1^. Different letters in each subcategory indicate statistical significant differences.

In total, 16 different manure types from different origins (including farmyard manure, i.e. various excretions originating from agricultural activity) were categorized of which seven were a combination of single manure types, which occurred in a low number and therefore were difficult to evaluate individually. The application of each manure type had a significantly positive response on SOC stocks (Figs. 5, 6). The lowest effects came from the application of green manure, straw, and combined applications of both with 17% and 5.1 Mg ha^−1^, 23% and 6.4 Mg ha^−1^ and 11% corresponding to 4.5 Mg ha^−1^, respectively. In contrast, pig manure, cattle manure and farmyard manure led to the highest responses with 50% and 15.8 Mg ha^−1^, 32% and 15 Mg ha^−1^ and 41% corresponding to 9.7 Mg ha^−1^, respectively. Other livestock excretions, namely poultry manure, sheep manure and horse manure responded with 39% and 8.9 Mg ha^−1^, 35% and 7 Mg ha^−1^ and 23% corresponding to 8.3 Mg ha^−1^, respectively, and thus also showed good SOC storage performances. Maillard and Angers^14^ found a comparable result with cattle manure, inducing high SOC stock differences, but they only considered three livestock species (cattle, pig, poultry) and they included only a small number of treatments, which led to a high variability. Liu et al*.*^40^ found an SOC response ratio of 12.8% after straw application on paddy and upland soils. This result corroborates our findings. Out of all manure types, pig manure showed the highest carbon accumulation potential. However, all manure types showed positive responses, especially those of livestock.

The effect of SOC stock changes influenced by additional added mineral fertilizer are presented in Figs. 5 and 6. If mineral fertilizer was added, the SOC stocks increased significantly by 40% and absolutely by 11.9 Mg ha^−1^. Treatments with no additional mineral fertilizer raised SOC stocks by 30%. Here, the absolute difference was slightly lower, with 10.2 Mg ha^−1^ (Fig. 6). As already explained in “General effect”, additional mineral fertilizer input provides a delivery of nutrients. Plant growth is promoted, aboveground and belowground. This enhanced net primary production with higher biomass inputs explains higher SOC stocks, as rising biomass yields generally correlate with rising SOC values. Although aboveground biomass is removed after harvest, increased root growth and higher crop residue amounts have a positive effect on SOC content, compared to unfertilized treatments, especially if manure and mineral fertilizer application is combined^49–51^. Further, also the relative SOC gain was higher if additional mineral fertilizer was used. Initial SOC values could be the explanation. The intercategorical evaluation of NPK and initial SOC values identified the most NPK treatments with low initial SOC values and high response ratios of 54% (Supplementary Material, Figure S13). Treatments with low initial SOC were also the majority in the non-NPK grouping, but many treatments with high SOC levels were also found. Here, response ratios only hardly differed from each other. ∆SOC showed the same dynamics for both NPK and non-NPK treatments with higher stock differences in soils (Supplementary Material, Figure S14).

**Figure 7:**
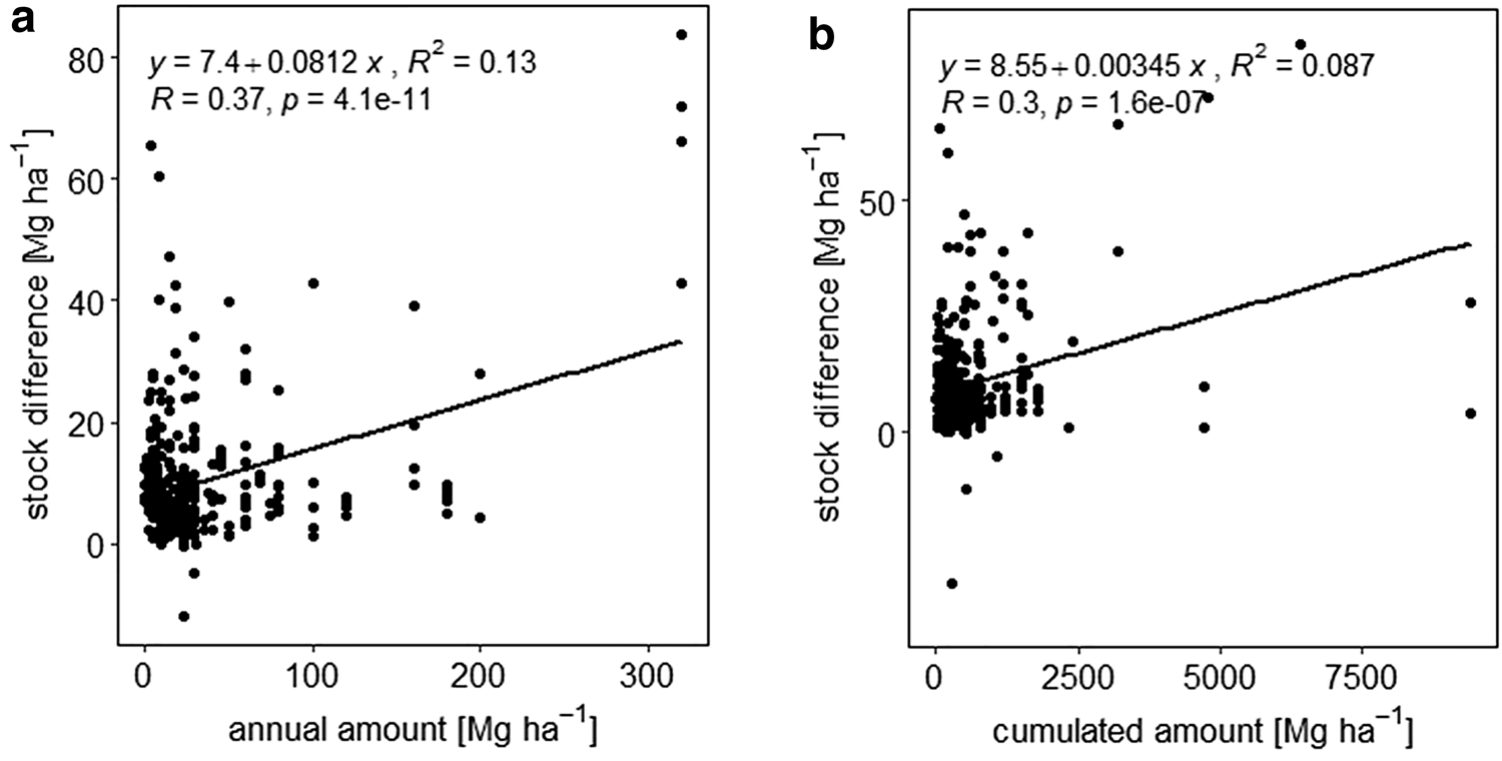
Relationship between the SOC stock difference (Mg ha^−1^) and cumulative manure-C input (Mg ha^−1^) (**a**) and the SOC stock difference (Mg ha^−1^) and annual manure input (Mg ha^−1^) (**b**). R^2^ represents the coefficient of determination.

SOC stock responses induced by different amounts of manure application are presented in Figs. 5 and 6. Cumulative and annual manure amounts, each in four different quantities were analyzed. Amounts < 500 Mg ha^−1^, the lowest cumulative quantity, showed the highest relative SOC stock response, which was 38%, but had a low absolute SOC gain, with 9.2 Mg ha^−1^ (Figs. 5, 6). The amounts ranging between 500 and 1000 Mg ha^−1^ showed a response ratio of 27% and a stock difference of 10.1 Mg ha^−1^. Between 1000 and 2000 Mg ha^−1^ the response was 30% relatively and 13.5 Mg ha^−1^ absolutely. The last and highest amount range classified was > 2000 Mg ha^−1^ with a response ratio of 19% and a 29.7 Mg ha^−1^ SOC stock change, which is the highest absolute value. Annual amount results showed a similar dynamic. Low annual manure amounts of 0–25 Mg ha^−1^ a^−1^ resulted in a low ∆SOC of 9.6 Mg ha^−1^ but a high relative change of 39%. High annual amounts > 100 Mg ha^−1^ a^−1^, however showed a high ∆SOC of 18.8 Mg ha^−1^ but a lower response ratio of 14%. The relative change in SOC stocks does not increase with higher annual input amounts. Rather, the response ratio reached the highest relative change at the lowest annual and cumulative input amount. However, our results indicate that high input amounts seem to be connected with high SOC stock differences. A regression analysis of the connection between the input amount and ∆SOC indicated a significant linear, but weak relationship for both annual (p = 4.1e−11; R^2^ = 0.13) (Fig. 7a) and cumulative quantities (p = 1.6e−07; R^2^ = 0.087) (Fig. 7b). Maillard and Angers^14^ also found a linear relationship between cumulative carbon input and SOC stock difference up to very high levels of carbon inputs which support our finding. To further evaluate this relationship, we analyzed the link between ∆SOC and annual manure inputs as a function of the subcategories we investigated. The regression plots are located in the Supplementary Material, Figure S15a–t. A Shapiro–Wilk test, which was carried out in advance, showed a non-normal distribution of the data. Regarding tillage intensity effects, no significant relationship could be found for reduced tillage treatments (Supplementary Material, Figure S15a), whereas conventional tillage treatments showed a linear relationship between annual amounts and ∆SOC (p < 2.2e−16; R^2^ = 0.5) (Supplementary Material, Figure S15b). Differences between soil texture groups could not be identified. All texture classes showed no significant relationships (Supplementary Material, Figure S15c—f). In the climate subcategories (Supplementary Material, Figure S15g,h), a significant relationship was only identified under non-tropical conditions (R^2^ = 0.24; p = 0.0019) (Supplementary Material, Figure S15g). Applications under tropical conditions were not included in the regression analysis due to the low number of treatments. Considering the various sampling depths (Supplementary Material, Figure S15i–l) only depths between 16 and 20 cm showed significance (R^2^ = 0.34; p = 3.1e−13) (Supplementary Material, Figure S15j). Regarding soil pH conditions (Supplementary Material, Figure S15m–o), a significant linear increase was identified in pH neutral soils but (R^2^ = 0.2; p = 9.9e-5) (Supplementary Material, Figure S15o), whereas acidic soils showed a significant linear decrease (R^2^ = 0.1; p = 0.044) (Supplementary Material, Figure S15m). Low initial SOC < 1% (R^2^ = 0.037; p = 0.049) (Supplementary Material, Figure S15p) and high initial SOC > 2% (R^2^ = 0.57; p = 1.6e−6) (Supplementary Material, Figure S15r) showed significant positive relationships. The application of additional mineral fertilizers led to an insignificant relation (Supplementary Material, Figure S15s) whereas non-NPK treatments showed a significant positive link (Supplementary Material, Figure S15t) (R^2^ = 0.41; p = 7.8e−15). To summarize, the annual amount of application seems to be important only under conventional tillage, non-tropical climate conditions and pH-neutral as well as SOC-rich or SOC-depleted soils and only if no additional mineral fertilization is applied. Under other conditions, there seems to be no statistically significant relation between ∆SOC and annual manure amounts.

## Conclusions

Globally, manure applications induced a raise of SOC stocks. However, our results indicate that the increase effect is linked to many factors and can show large differences. These factors included management decisions (tillage intensity, manure amount, duration of application), site properties (climate, initial SOC content, soil texture) and manure characteristics (manure origin and the combined application with synthetic fertilizer). To better understand carbon dynamics, more long-term SOC field data are required, especially the factors influencing carbon saturation need to be further investigated. Moreover, many measurements under tropical conditions need to be conducted because the small number of treatments found made it impossible to draw definitive conclusions. Additional to that, more holistic approaches within carbon dynamics assessment methods need to be established. For example, although, conventional tillage and synthetic fertilization have high effects in terms of SOC enrichment, positive aspects through reduced tillage and external effects (e.g. through the production of synthetic fertilizers) should play a role in the development of sustainable management strategies. Expanding the scope will help to avoid misleading conclusions.

## Supplementary Information

Supplementary Material

Supplementary Datasets
