# Supplementary material for: Meta-analysis on how manure application changes soil organic carbon storage
Source: Sci Rep. 2021 Mar 9;11:5516. doi: 10.1038/s41598-021-82739-7 (PMC7943820; doi:10.1038/s41598-021-82739-7)
Supplement: Supplementary file 1 — Supplementary Material [file 41598_2021_82739_MOESM1_ESM.docx]

**Meta – analysis on how manure application changes soil organic carbon storage**

Authors: Arthur Gross and Bruno Glaser

Supplementary Material

Meta-analysis results of the intercategorical grouping


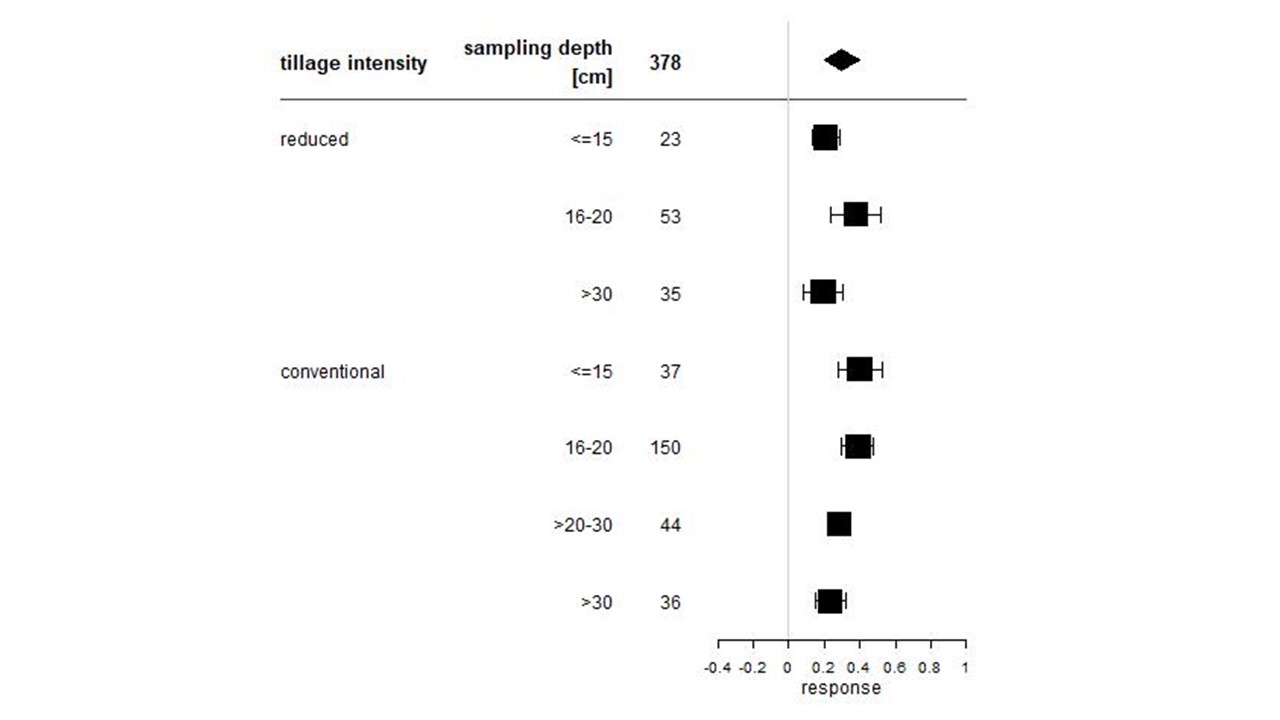


**Figure S1.** Relative response of manure applications on soil organic carbon stocks influenced by the tillage intensity in different sampling depths of the considered treatments. The overall grand mean of all individual treatments is presented in the first row followed by the considered subcategories below. Each response ratio is presented as the range between the upper and lower 95% confidence intervals. Points within the range represent the mean response ratio. The range between both 95% confidence intervals of the grand mean is shown by the extent of the rectangle. The number in each treatment row represents the number of pairwise comparisons on which the statistic is based. The grey line was drawn at response ratio = 0. Different letters in each subcategory indicate statistical significant differences.

**
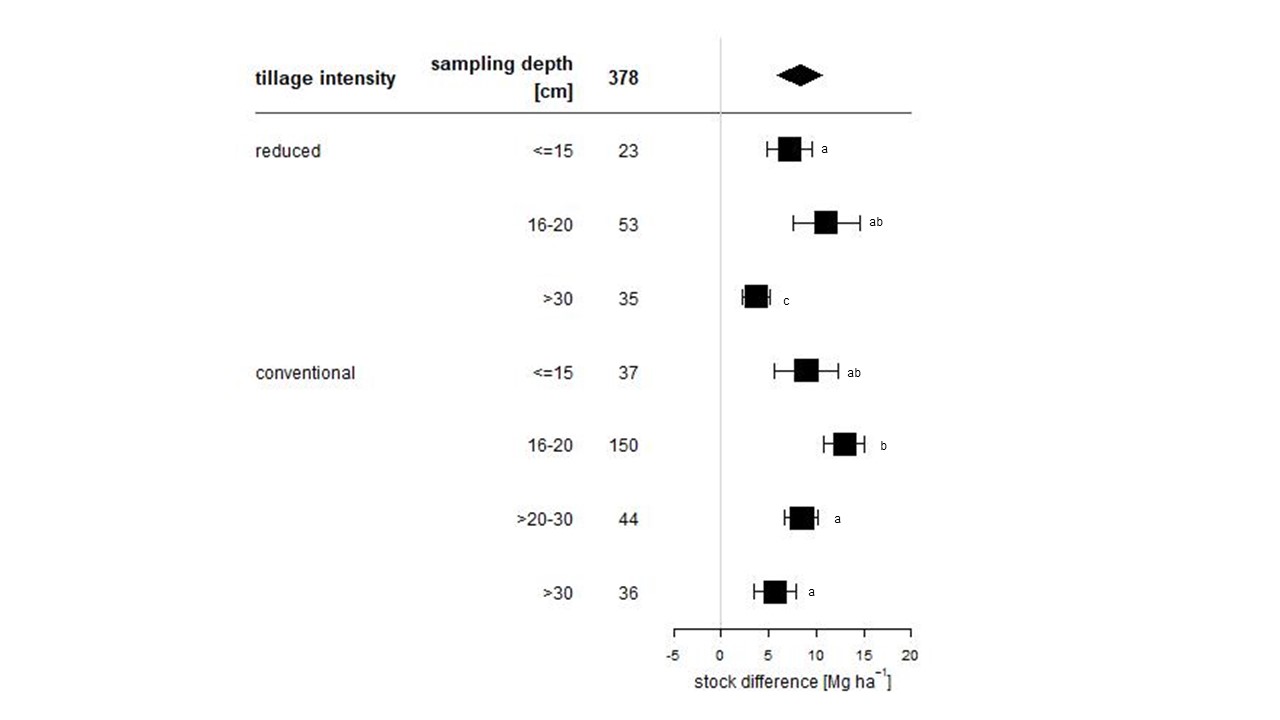
Figure S2.** Absolute response (Mg ha^-1^) of manure applications on soil organic carbon stocks influenced by the tillage intensity in different sampling depths of the considered treatments. The overall grand mean of all individual treatments is presented in the first row followed by the considered subcategories below. Each response ratio is presented as the range between the upper and lower 95% confidence intervals. Points within the range represent the mean response ratio. The range between both 95% confidence intervals of the grand mean is shown by the extent of the rectangle. The number in each treatment row represents the number of pairwise comparisons on which the statistic is based. The grey line was drawn at stock difference = 0 Mg ha^-1^. Different letters in each subcategory indicate statistical significant differences.

**
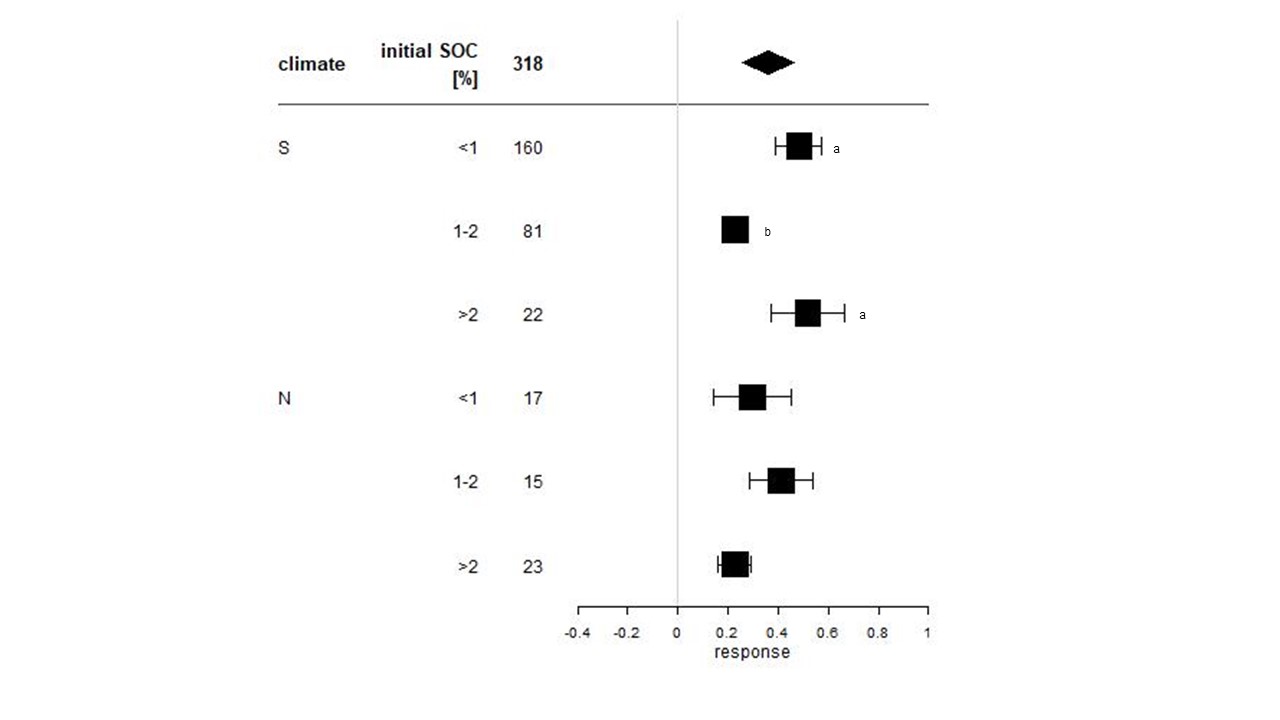
**

**Figure S3.** Relative response of manure applications on soil organic carbon stocks influenced by the combined effect of the climate and different initial soil organic carbon contents (%) of the considered treatments. The overall grand mean of all individual treatments is presented in the first row followed by the considered subcategories below. Each response ratio is presented as the range between the upper and lower 95% confidence intervals. Points within the range represent the mean response ratio. The range between both 95% confidence intervals of the grand mean is shown by the extent of the rectangle. The number in each treatment row represents the number of pairwise comparisons on which the statistic is based. The grey line was drawn at response ratio = 0. Different letters in each subcategory indicate statistical significant differences.

**
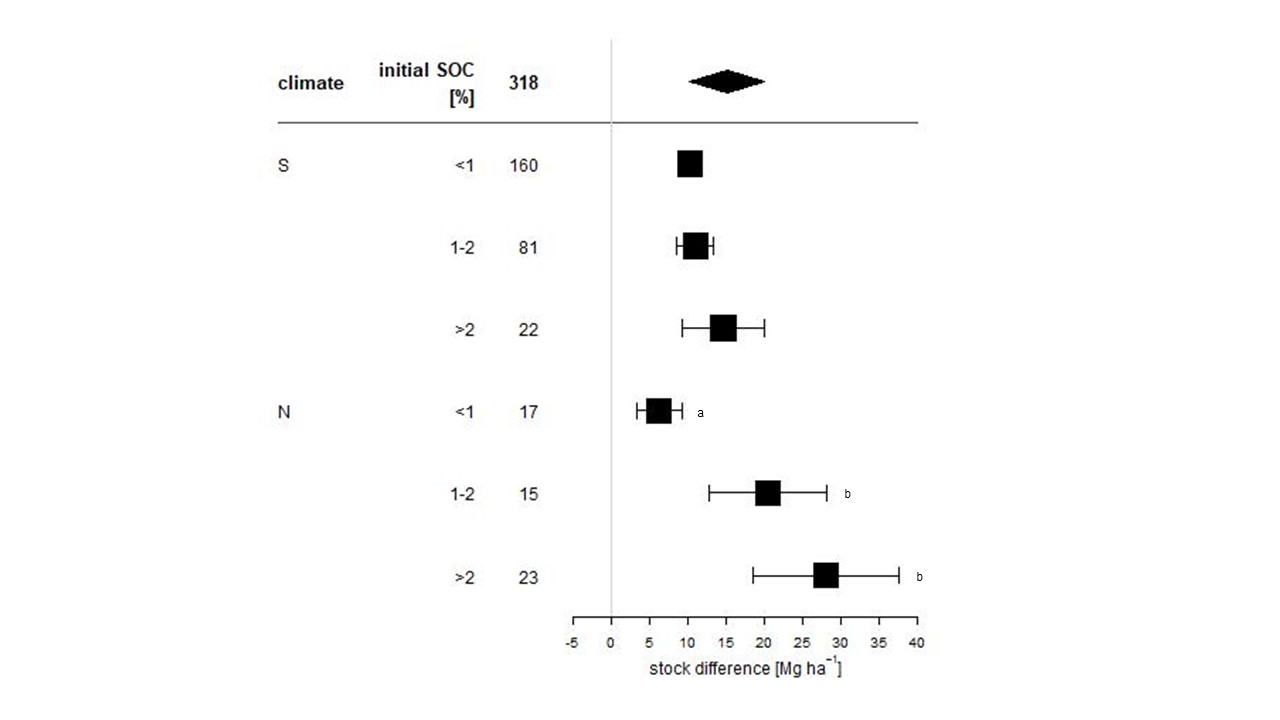
Figure S4.** Absolute response (Mg ha^-1^) of manure applications on soil organic carbon stocks influenced by the combined effect of climate and different initial soil organic carbon contents (%) of the considered treatments. The overall grand mean of all individual treatments is presented in the first row followed by the considered subcategories below. Each response ratio is presented as the range between the upper and lower 95% confidence intervals. Points within the range represent the mean response ratio. The range between both 95% confidence intervals of the grand mean is shown by the extent of the rectangle. The number in each treatment row represents the number of pairwise comparisons on which the statistic is based. The grey line was drawn at stock difference = 0 Mg ha^-1^. Different letters in each subcategory indicate statistical significant differences.

**
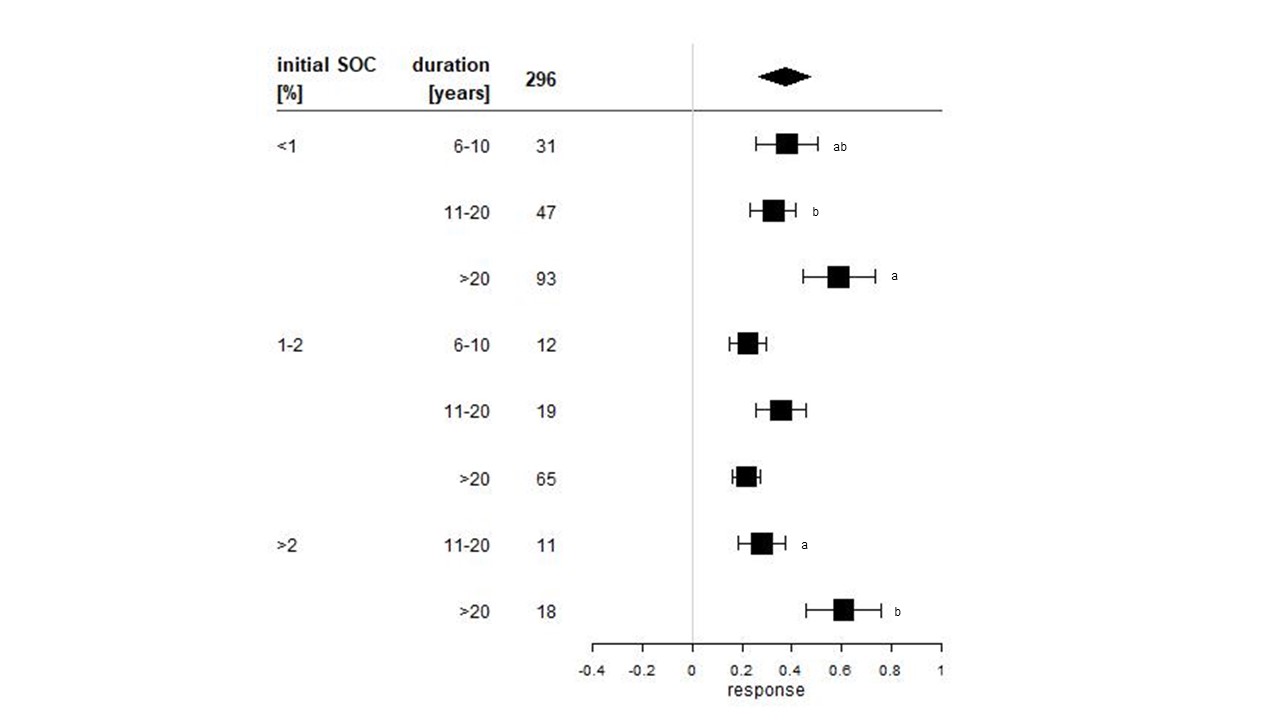
Figure S5.** Relative response of manure applications on soil organic carbon stocks influenced by the combined effect of the initial soil organic carbon content (%) and different experiment durations of the considered treatments. The overall grand mean of all individual treatments is presented in the first row followed by the considered subcategories below. Each response ratio is presented as the range between the upper and lower 95% confidence intervals. Points within the range represent the mean response ratio. The range between both 95% confidence intervals of the grand mean is shown by the extent of the rectangle. The number in each treatment row represents the number of pairwise comparisons on which the statistic is based. The grey line was drawn at response ratio = 0. Different letters in each subcategory indicate statistical significant differences.

**
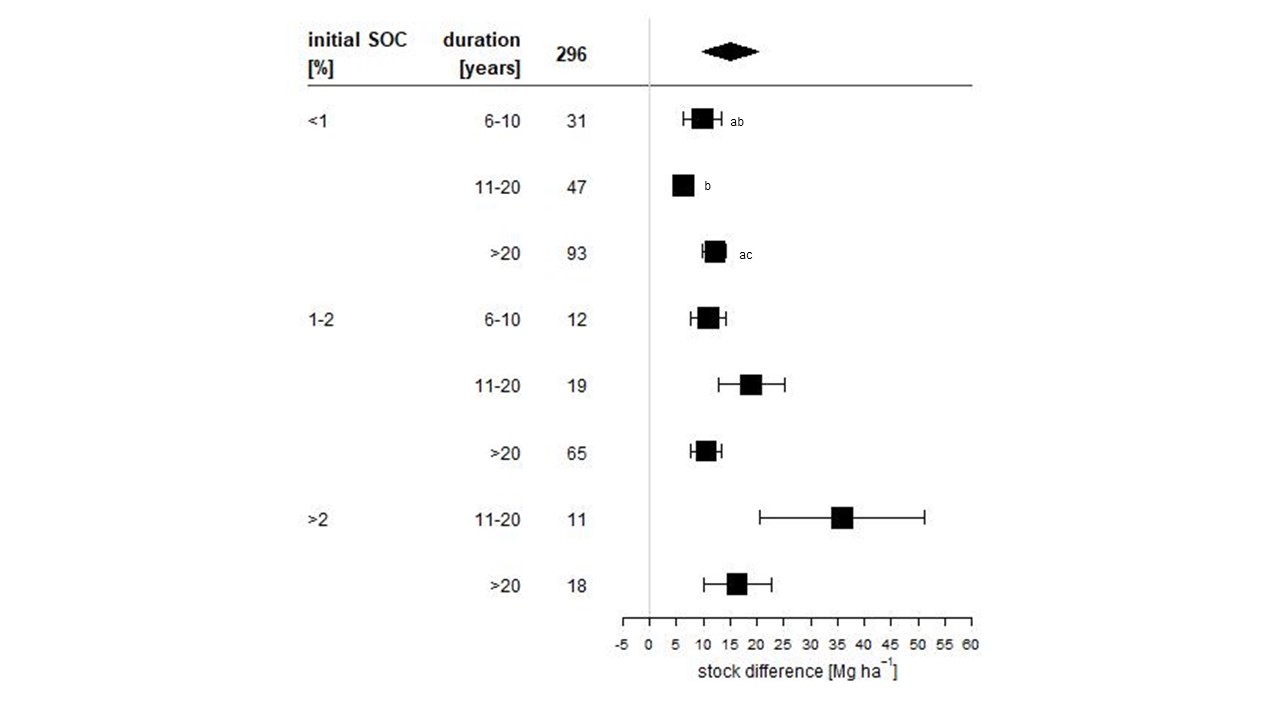
Figure S6.** Absolute response (Mg ha^-1^) of manure applications on soil organic carbon stocks influenced by the combined effect of the initial soil organic carbon content (%) and different experiment durations of the considered treatments. The overall grand mean of all individual treatments is presented in the first row followed by the considered subcategories below. Each response ratio is presented as the range between the upper and lower 95% confidence intervals. Points within the range represent the mean response ratio. The range between both 95% confidence intervals of the grand mean is shown by the extent of the rectangle. The number in each treatment row represents the number of pairwise comparisons on which the statistic is based. The grey line was drawn at stock difference = 0 Mg ha^-1^. Different letters in each subcategory indicate statistical significant differences.

**
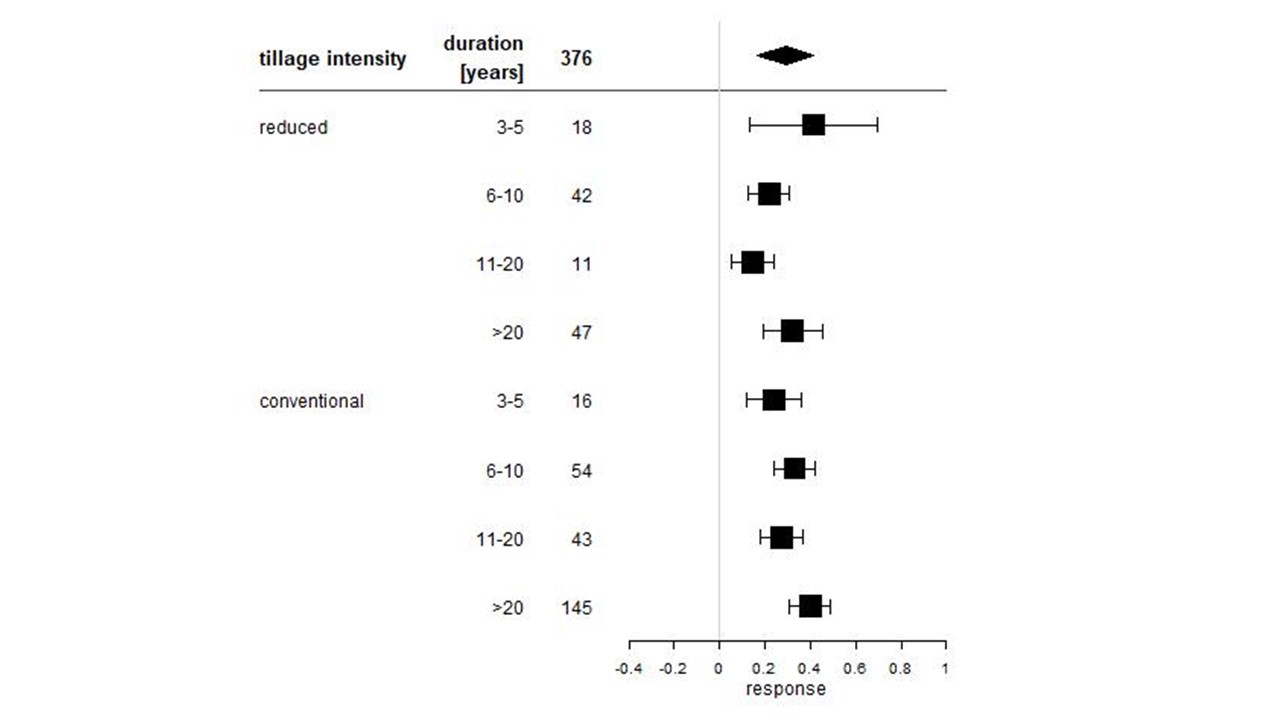
Figure S7.** Relative response of manure applications on soil organic carbon stocks influenced by the combined effect of the tillage intensity and the experiment duration of the considered treatments. The overall grand mean of all individual treatments is presented in the first row followed by the considered subcategories below. Each response ratio is presented as the range between the upper and lower 95% confidence intervals. Points within the range represent the mean response ratio. The range between both 95% confidence intervals of the grand mean is shown by the extent of the rectangle. The number in each treatment row represents the number of pairwise comparisons on which the statistic is based. The grey line was drawn at response ratio = 0. Different letters in each subcategory indicate statistical significant differences.

**
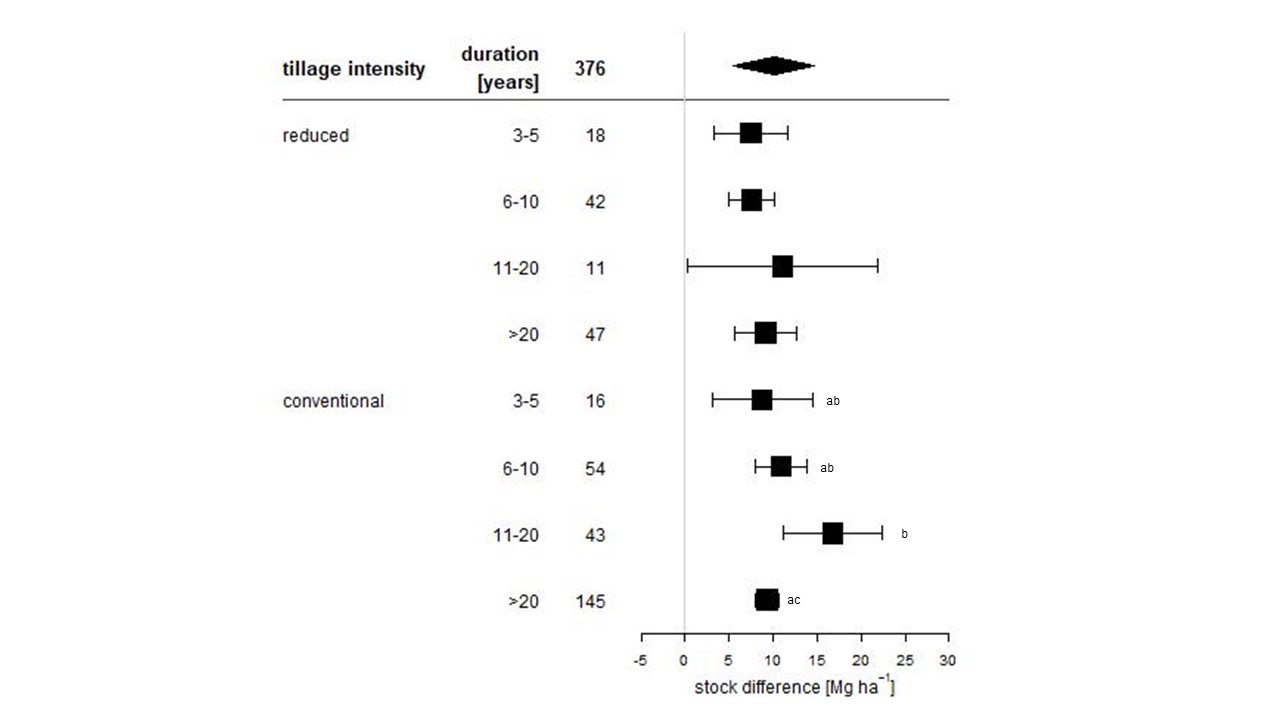
Figure S8.** Absolute response (Mg ha^-1^) of manure applications on soil organic carbon stocks influenced by the combined effect of the tillage intensity and the experiment duration of the considered treatments. The overall grand mean of all individual treatments is presented in the first row followed by the considered subcategories below. Each response ratio is presented as the range between the upper and lower 95% confidence intervals. Points within the range represent the mean response ratio. The range between both 95% confidence intervals of the grand mean is shown by the extent of the rectangle. The number in each treatment row represents the number of pairwise comparisons on which the statistic is based. The grey line was drawn at stock difference = 0 Mg ha^-1^. Different letters in each subcategory indicate statistical significant differences.

**
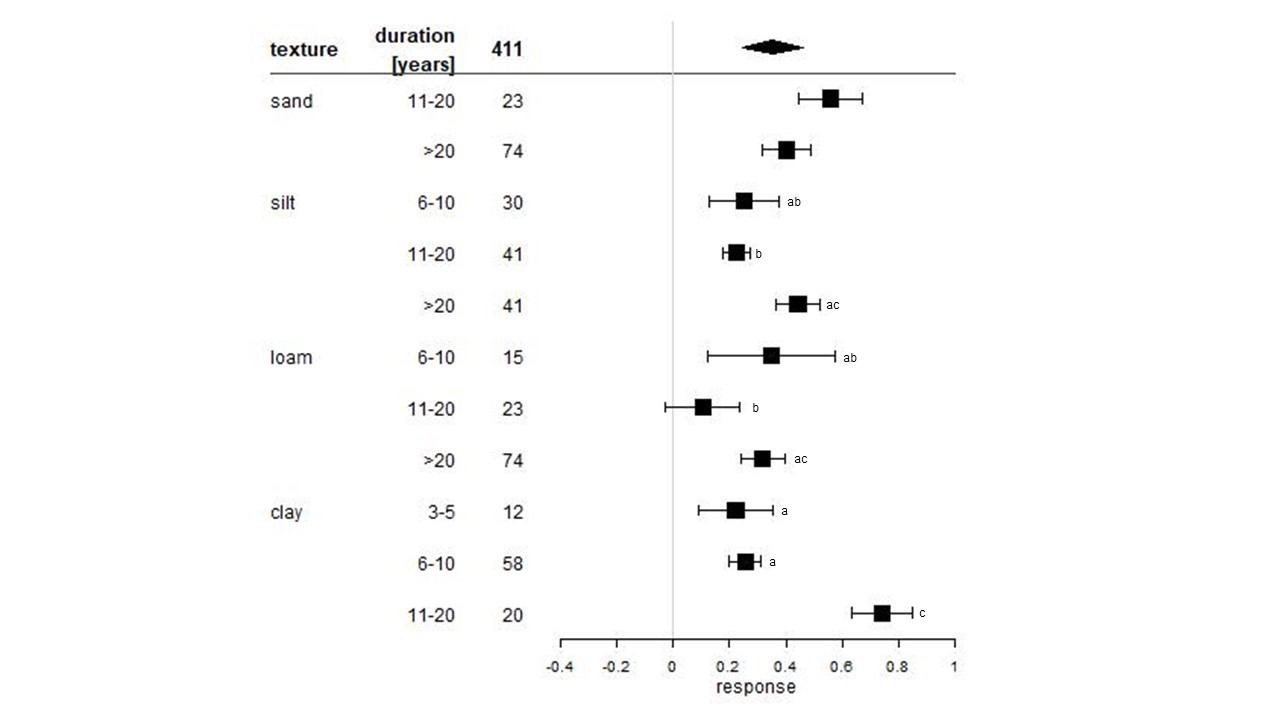
Figure S9.** Relative response of manure applications on soil organic carbon stocks influenced by the combined effect of different soil textures and the experiment duration of the considered treatments. The overall grand mean of all individual treatments is presented in the first row followed by the considered subcategories below. Each response ratio is presented as the range between the upper and lower 95% confidence intervals. Points within the range represent the mean response ratio. The range between both 95% confidence intervals of the grand mean is shown by the extent of the rectangle. The number in each treatment row represents the number of pairwise comparisons on which the statistic is based. The grey line was drawn at response ratio = 0. Different letters in each subcategory indicate statistical significant differences.

**
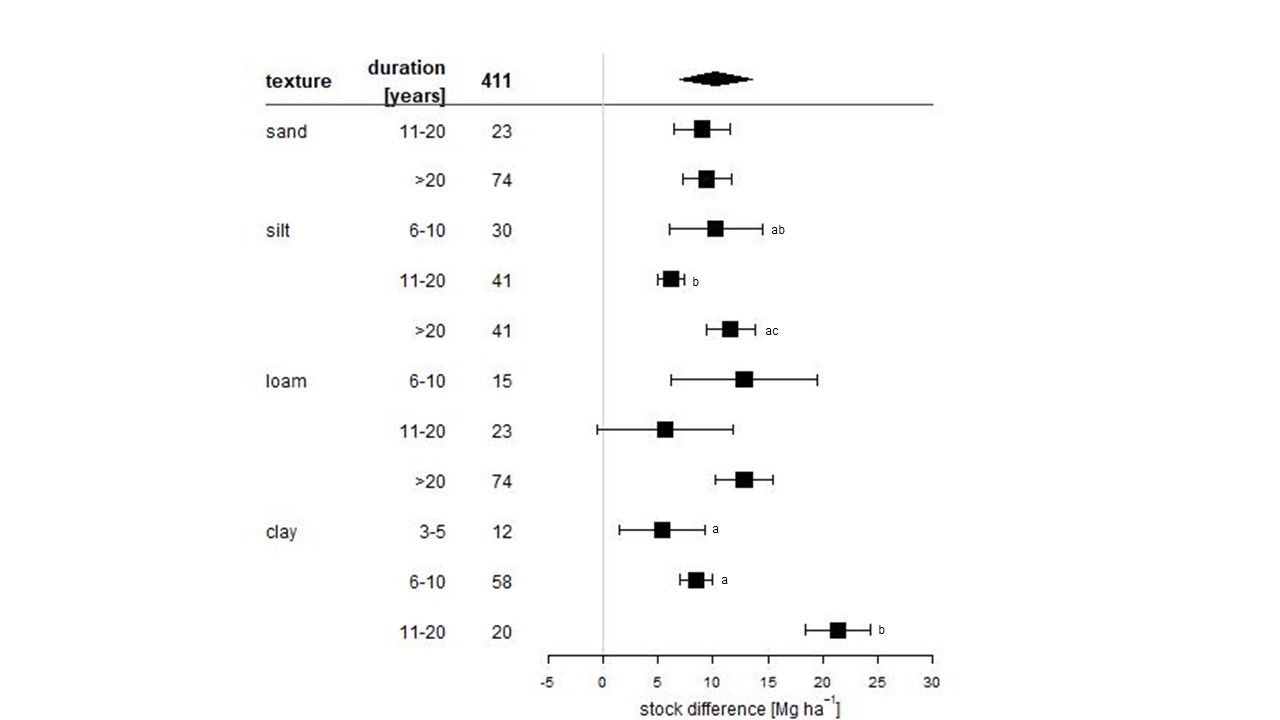
Figure S10.** Absolute response (Mg ha^-1^) of manure applications on soil organic carbon stocks influenced by the combined effect of different soil textures and the experiment duration of the considered treatments. The overall grand mean of all individual treatments is presented in the first row followed by the considered subcategories below. Each response ratio is presented as the range between the upper and lower 95% confidence intervals. Points within the range represent the mean response ratio. The range between both 95% confidence intervals of the grand mean is shown by the extent of the rectangle. The number in each treatment row represents the number of pairwise comparisons on which the statistic is based. The grey line was drawn at stock difference = 0 Mg ha^-1^. Different letters in each subcategory indicate statistical significant differences.

**
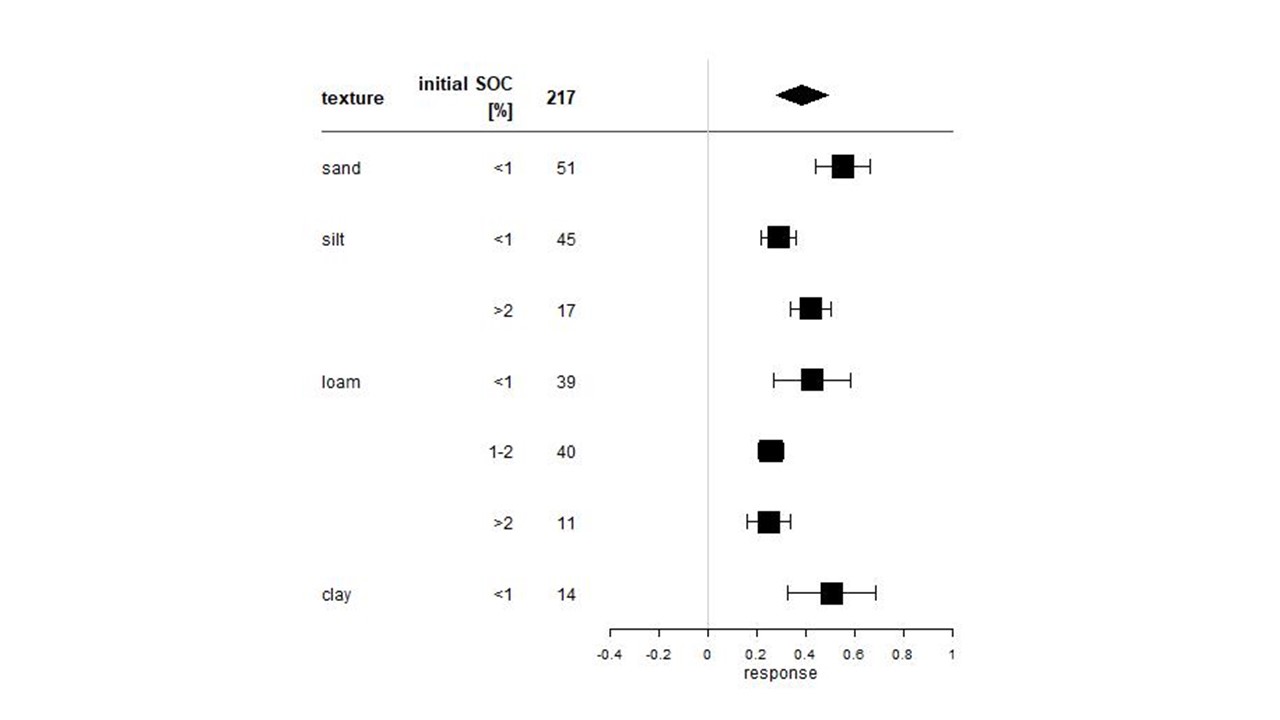
Figure S11.** Relative response of manure applications on soil organic carbon stocks influenced by the combined effect of different soil textures and the initial soil organic carbon content (%) of the considered treatments. The overall grand mean of all individual treatments is presented in the first row followed by the considered subcategories below. Each response ratio is presented as the range between the upper and lower 95% confidence intervals. Points within the range represent the mean response ratio. The range between both 95% confidence intervals of the grand mean is shown by the extent of the rectangle. The number in each treatment row represents the number of pairwise comparisons on which the statistic is based. The grey line was drawn at response ratio = 0. Different letters in each subcategory indicate statistical significant differences.

**
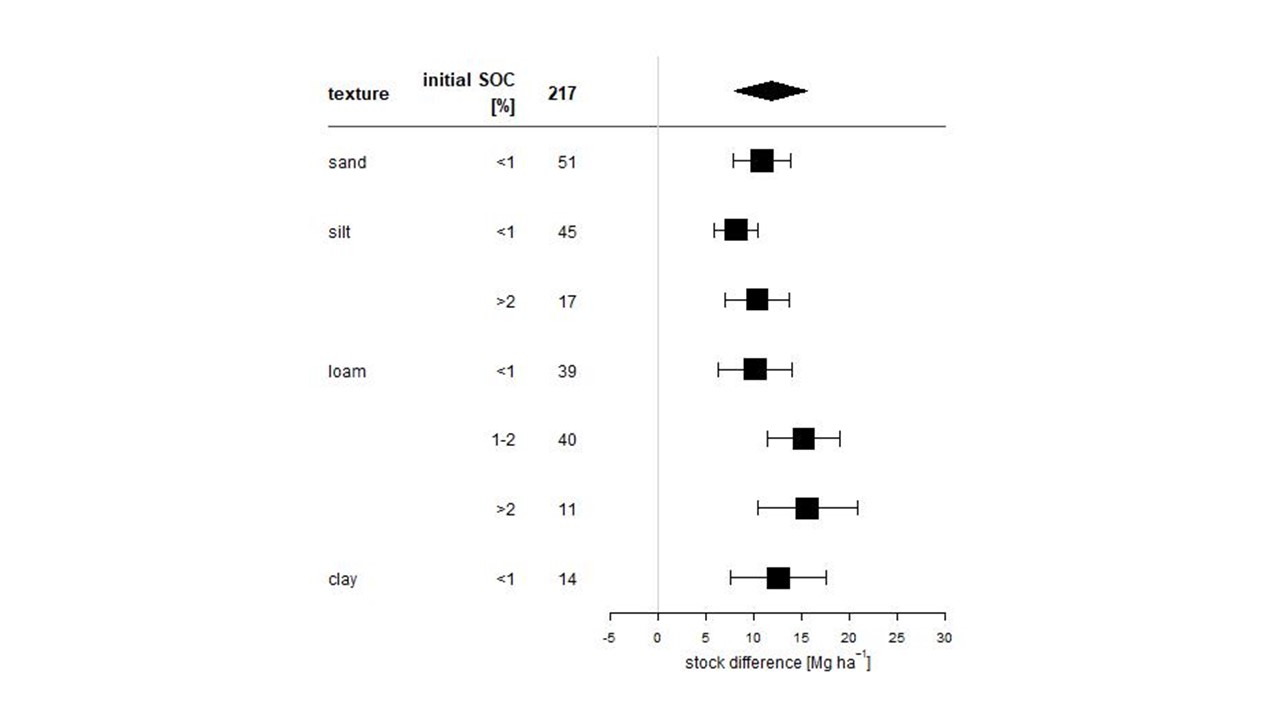
Figure S12.** Absolute response (Mg ha^-1^) of manure applications on soil organic carbon stocks influenced by the combined effect of different soil textures and the initial soil organic carbon content (%) of the considered treatments. The overall grand mean of all individual treatments is presented in the first row followed by the considered subcategories below. Each response ratio is presented as the range between the upper and lower 95% confidence intervals. Points within the range represent the mean response ratio. The range between both 95% confidence intervals of the grand mean is shown by the extent of the rectangle. The number in each treatment row represents the number of pairwise comparisons on which the statistic is based. The grey line was drawn at stock difference = 0 Mg ha^-1^. Different letters in each subcategory indicate statistical significant differences.

**
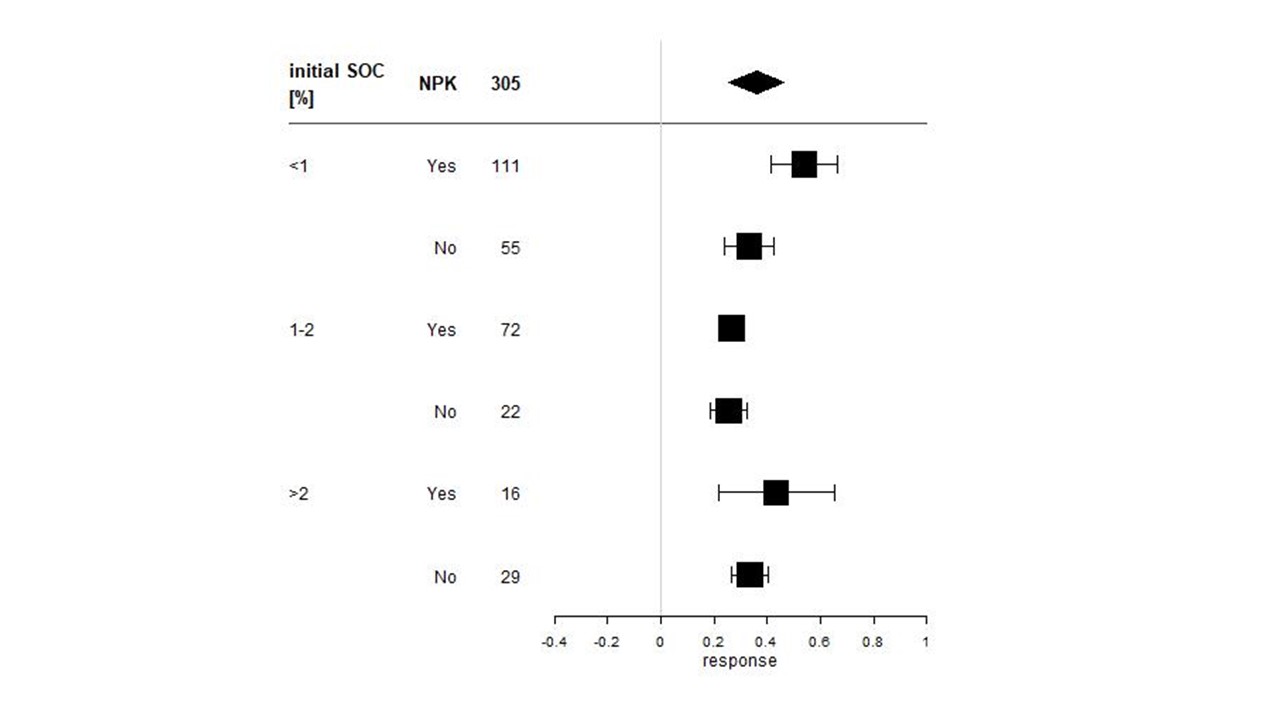
**

**Figure S13.** Relative response of manure applications on soil organic carbon stocks influenced by the combined effect of the initial soil organic carbon content (%) and additional added chemical fertilizer (NPK) of the considered treatments. The overall grand mean of all individual treatments is presented in the first row followed by the considered subcategories below. Each response ratio is presented as the range between the upper and lower 95% confidence intervals. Points within the range represent the mean response ratio. The range between both 95% confidence intervals of the grand mean is shown by the extent of the rectangle. The number in each treatment row represents the number of pairwise comparisons on which the statistic is based. The grey line was drawn at response ratio = 0. Different letters in each subcategory indicate statistical significant differences.

**
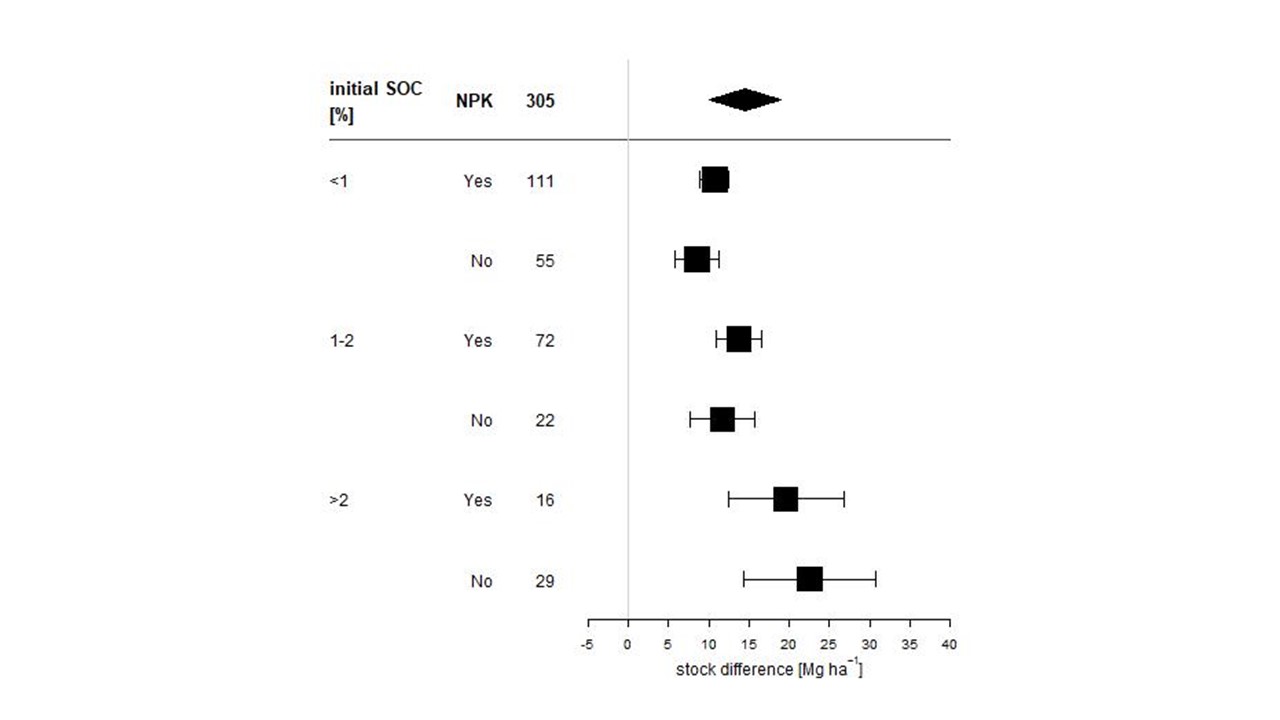
Figure S14.** Absolute response (Mg ha^-1^) of manure applications on soil organic carbon stocks influenced by the combined effect of the initial soil organic carbon content (%) and additional added chemical fertilizer (NPK) of the considered treatments. The overall grand mean of all individual treatments is presented in the first row followed by the considered subcategories below. Each response ratio is presented as the range between the upper and lower 95% confidence intervals. Points within the range represent the mean response ratio. The range between both 95% confidence intervals of the grand mean is shown by the extent of the rectangle. The number in each treatment row represents the number of pairwise comparisons on which the statistic is based. The grey line was drawn at stock difference = 0 Mg ha^-1^. Different letters in each subcategory indicate statistical significant differences.

**a)**

**
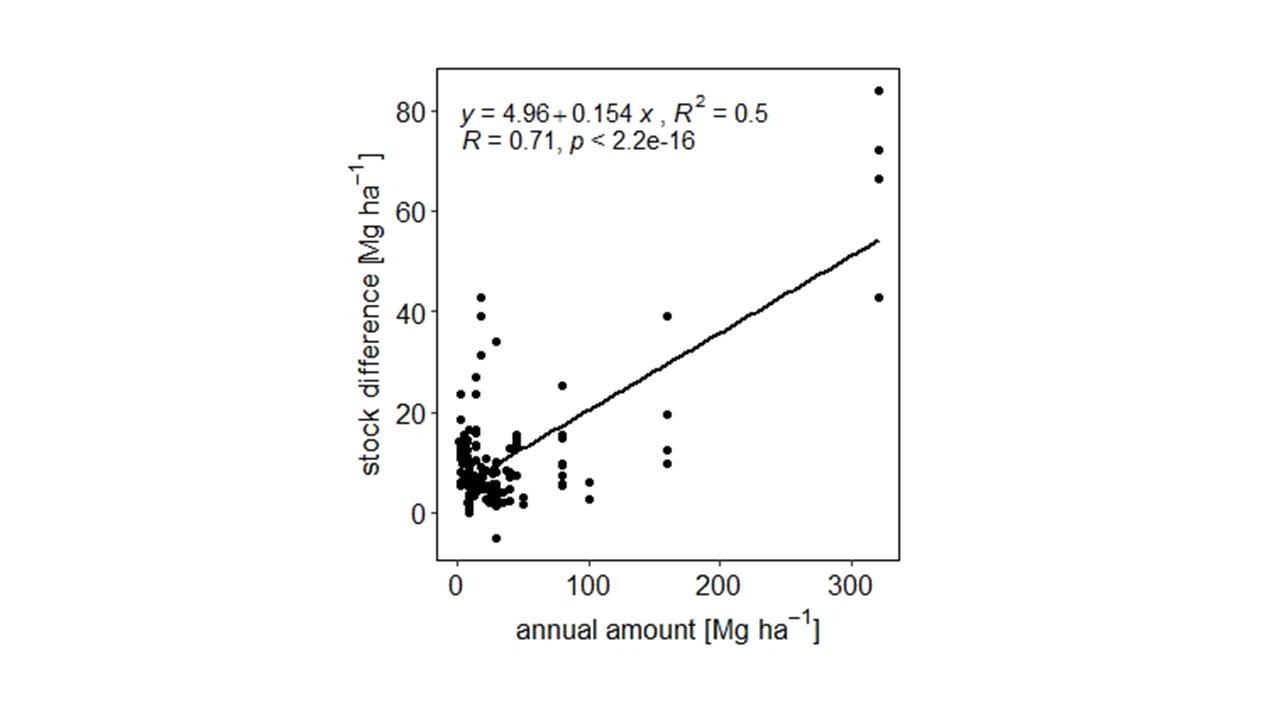
**

**b)**

**
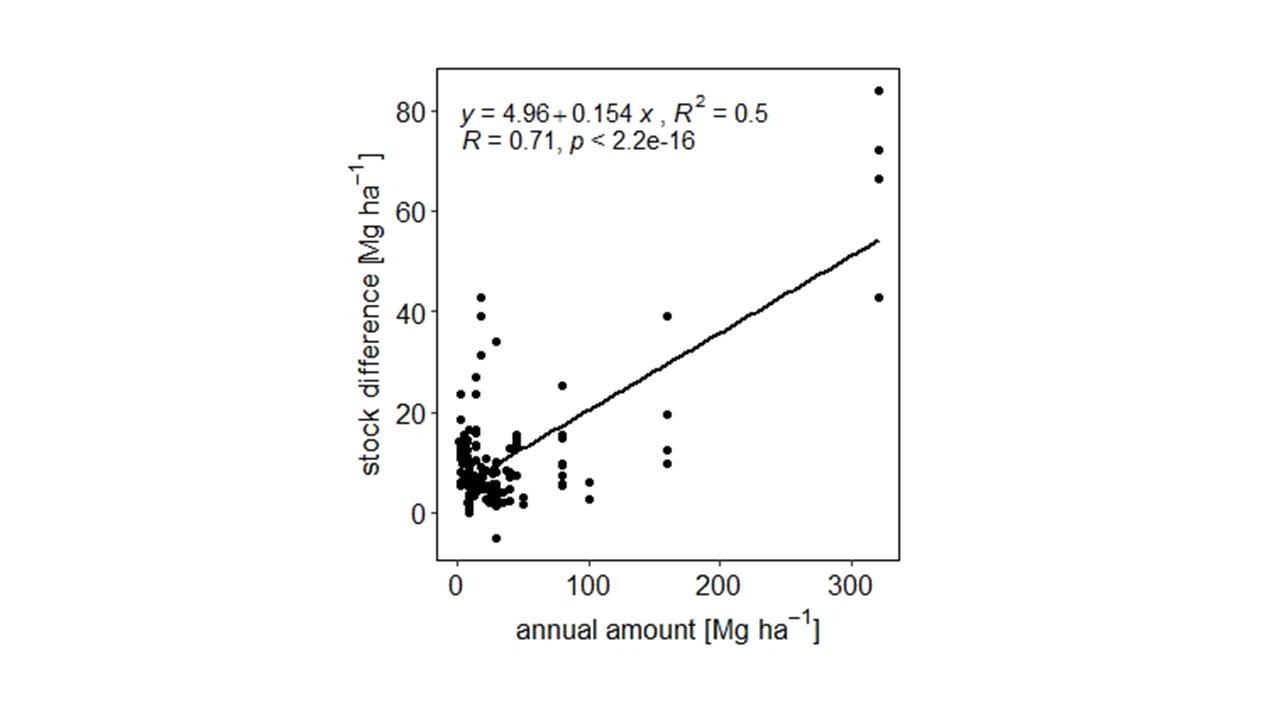
**

**c)**

**
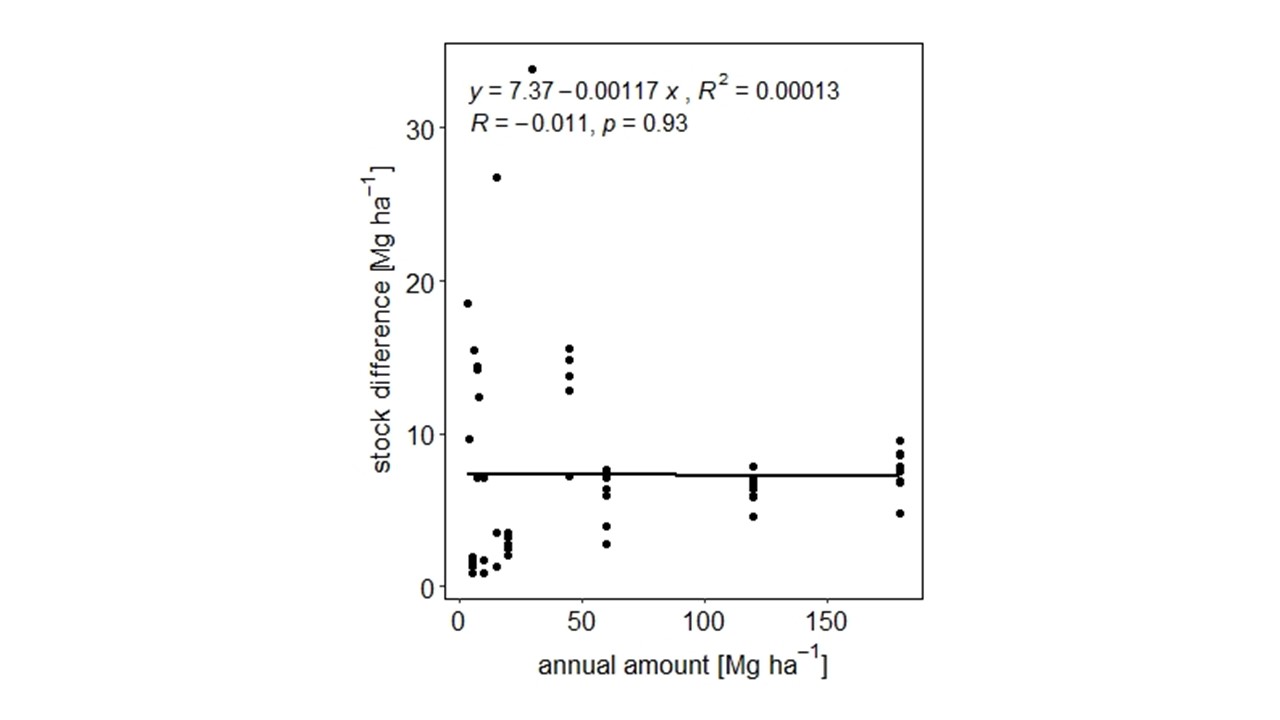
**

**d)**

**
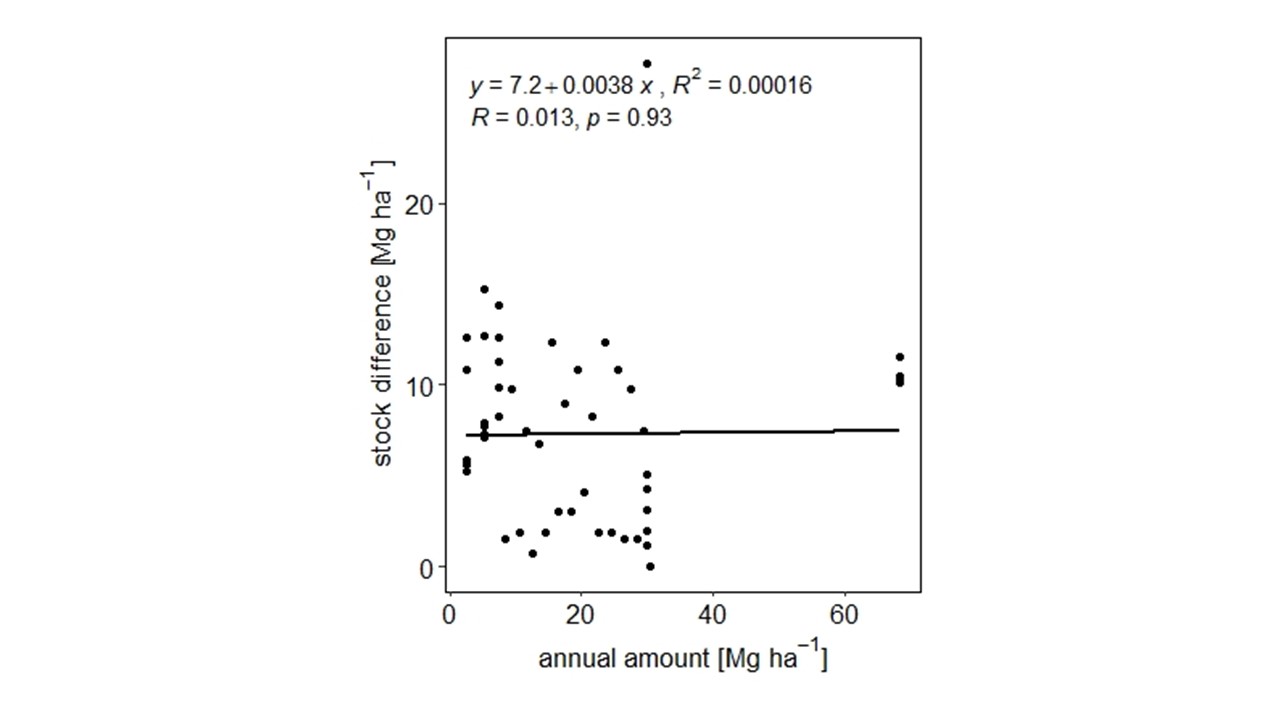
**

**e)**

**
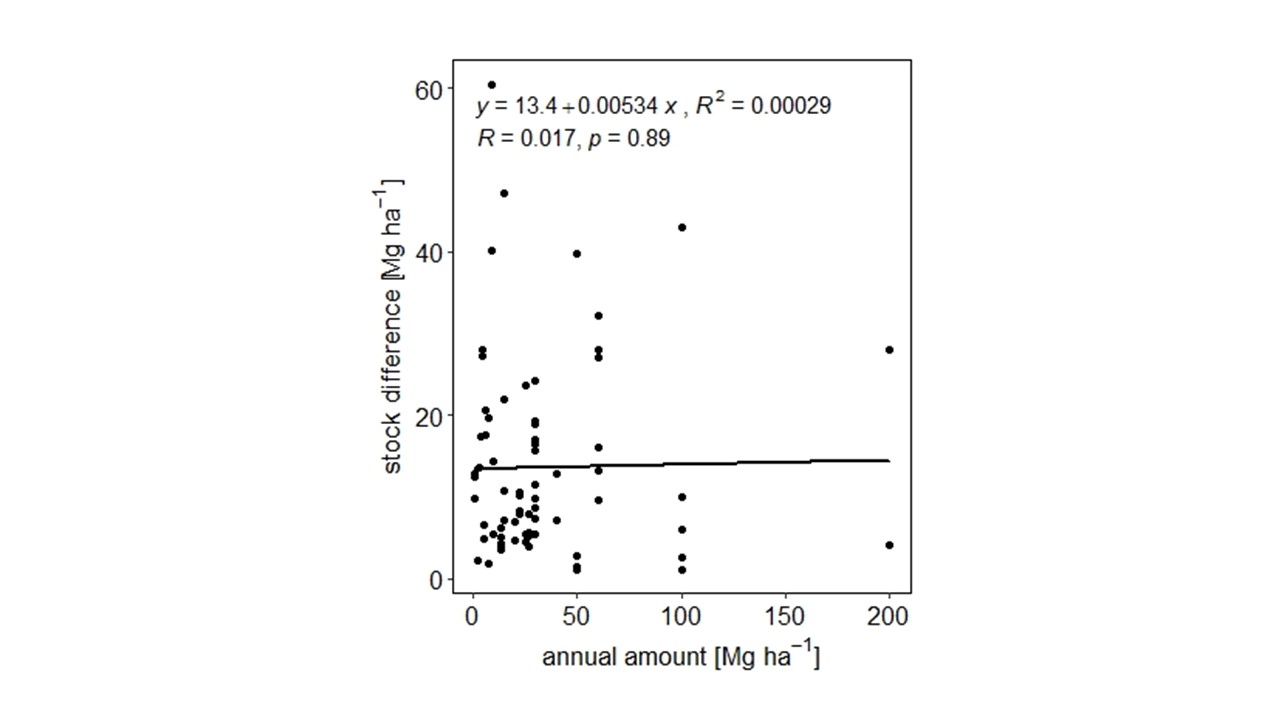
**

**f)**

**
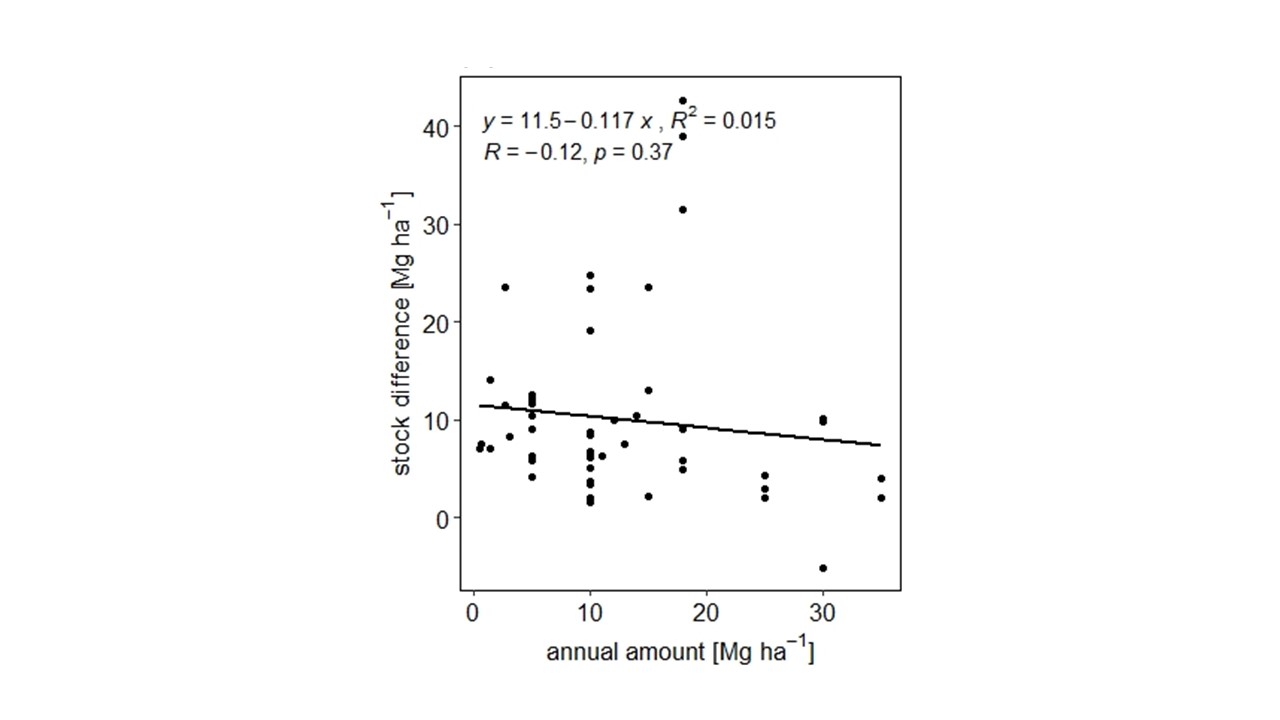
**

**g)**

**
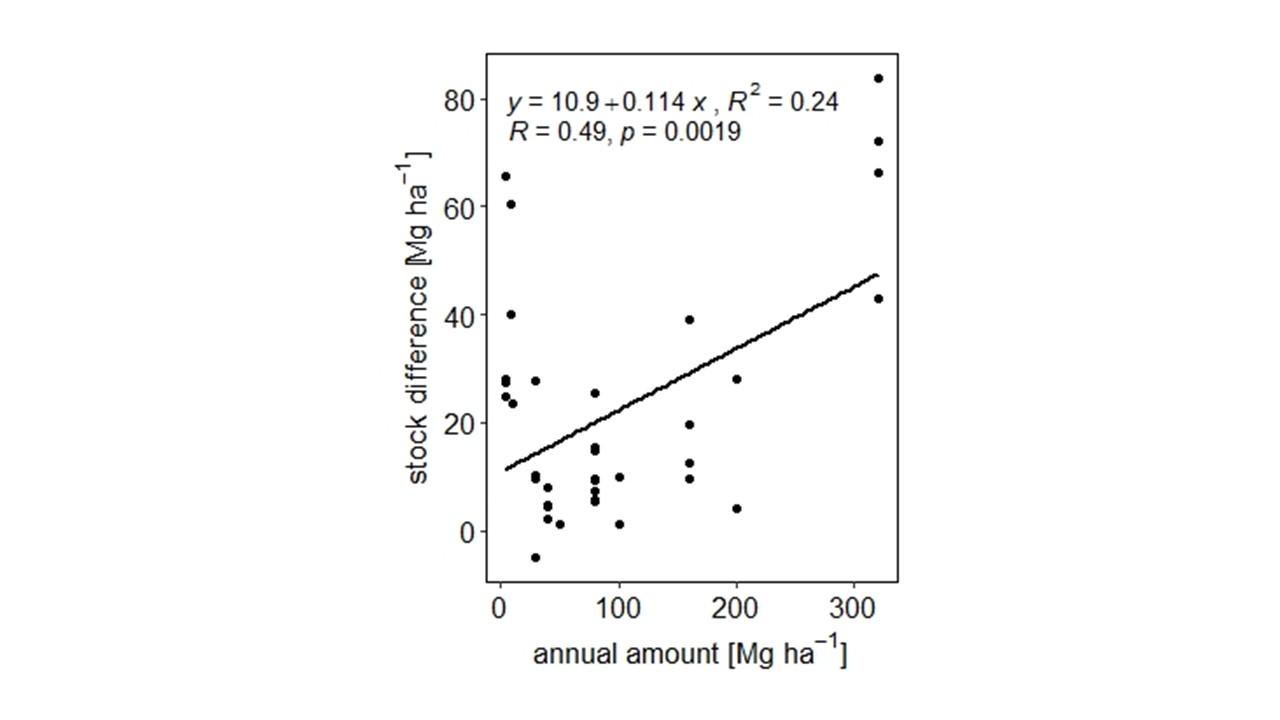
**

**h)**

**
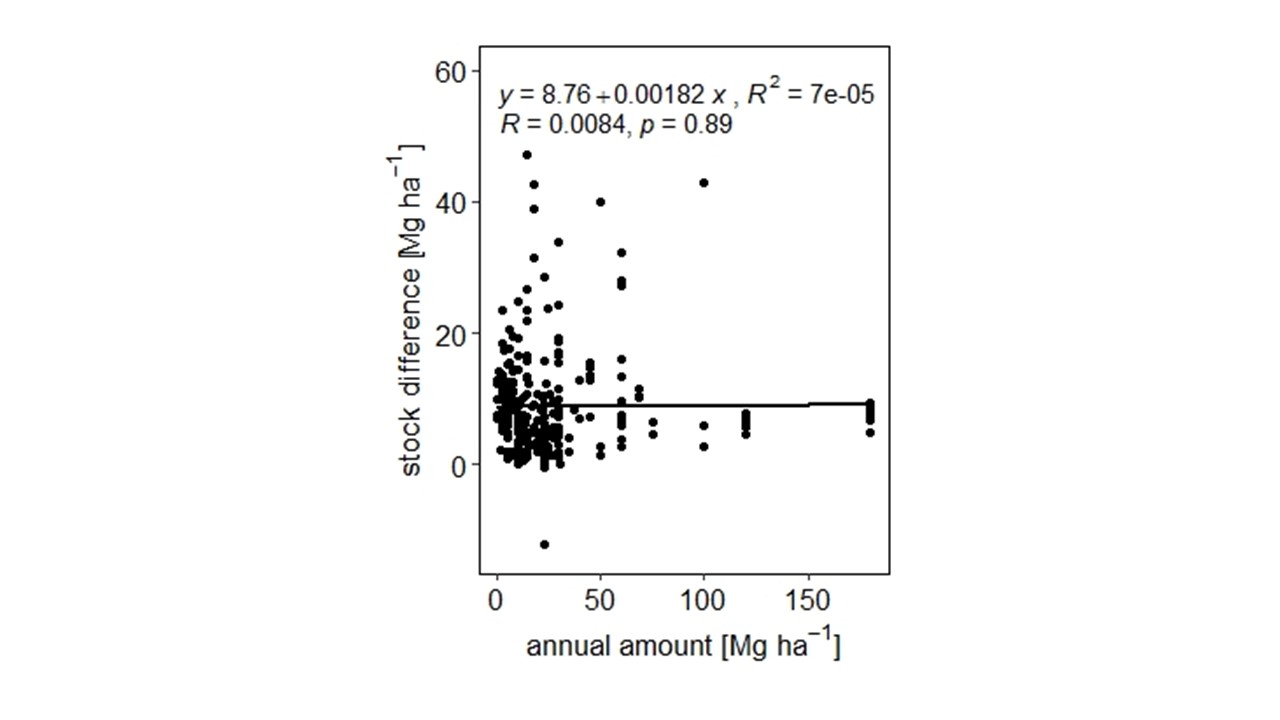
**

**i)**

**
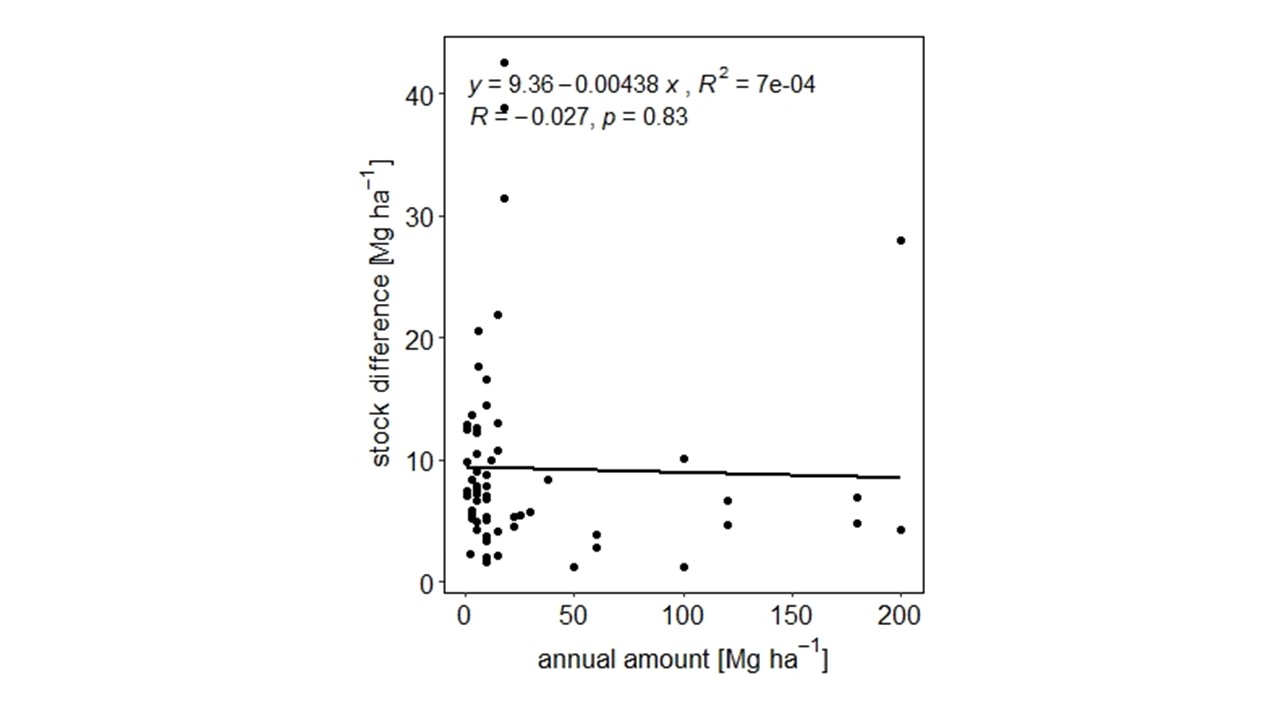
**

**j)**

**
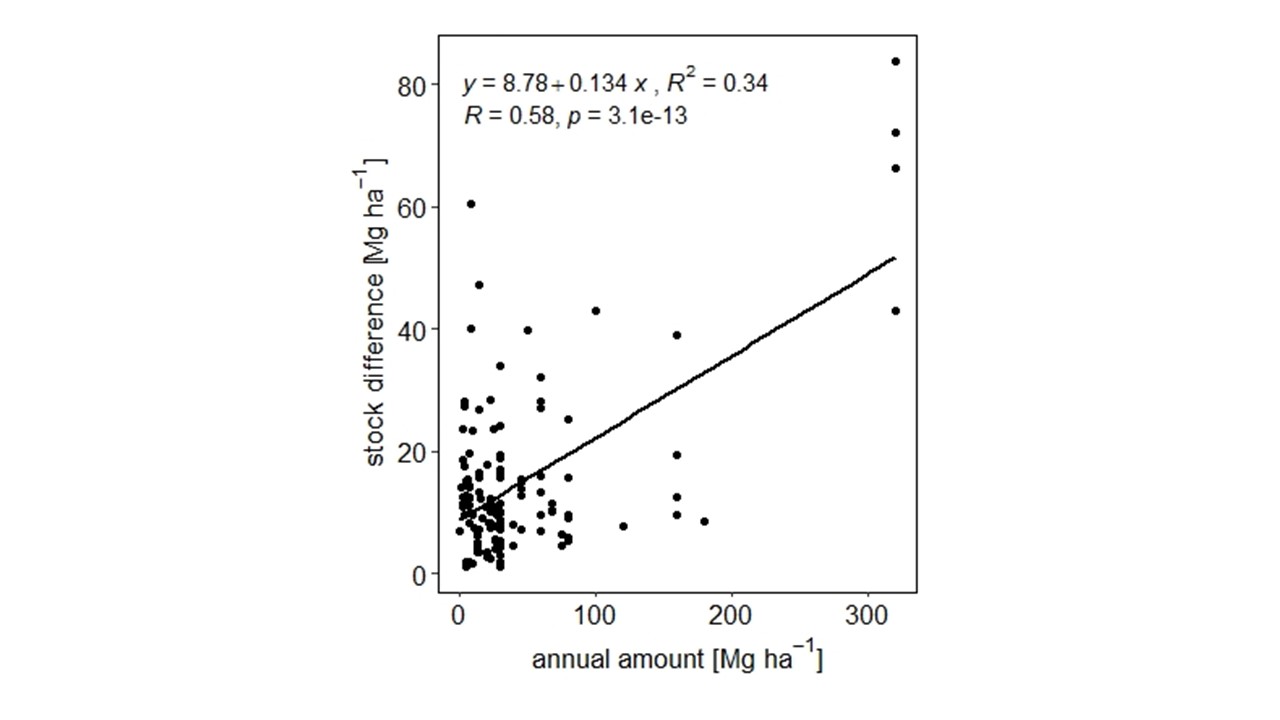
**

**k)**

**
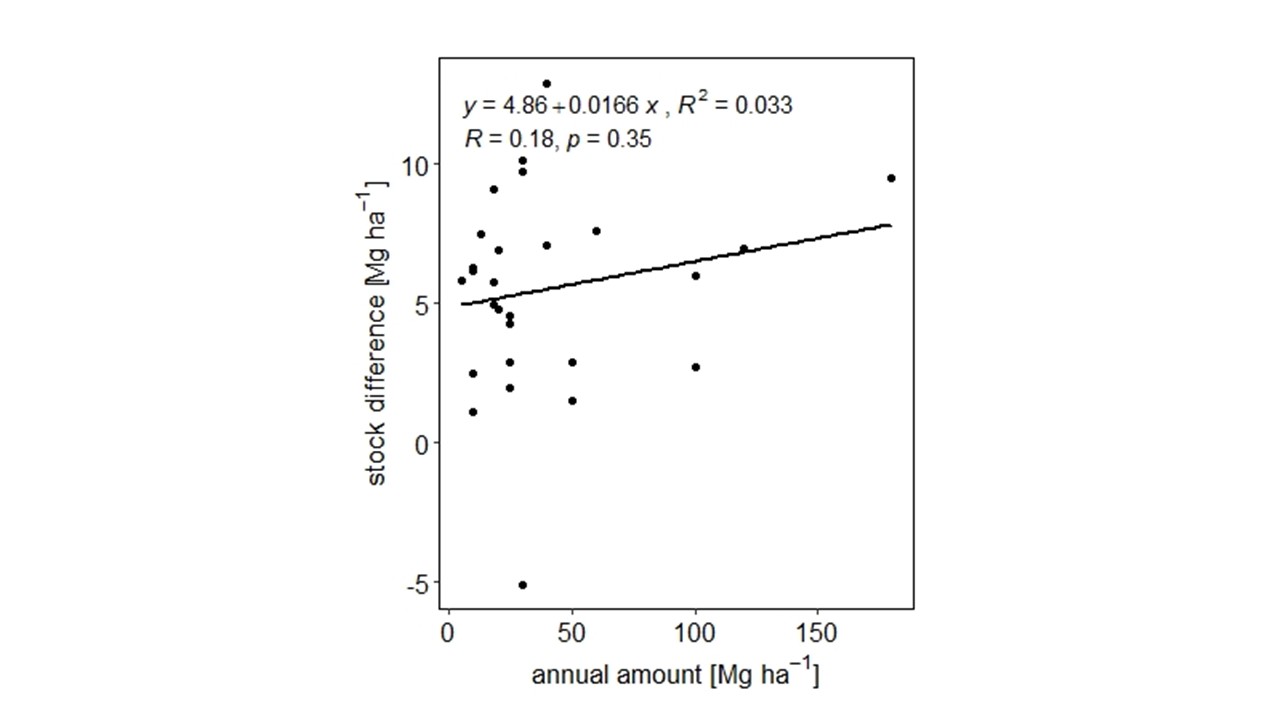
**

**l)**

**
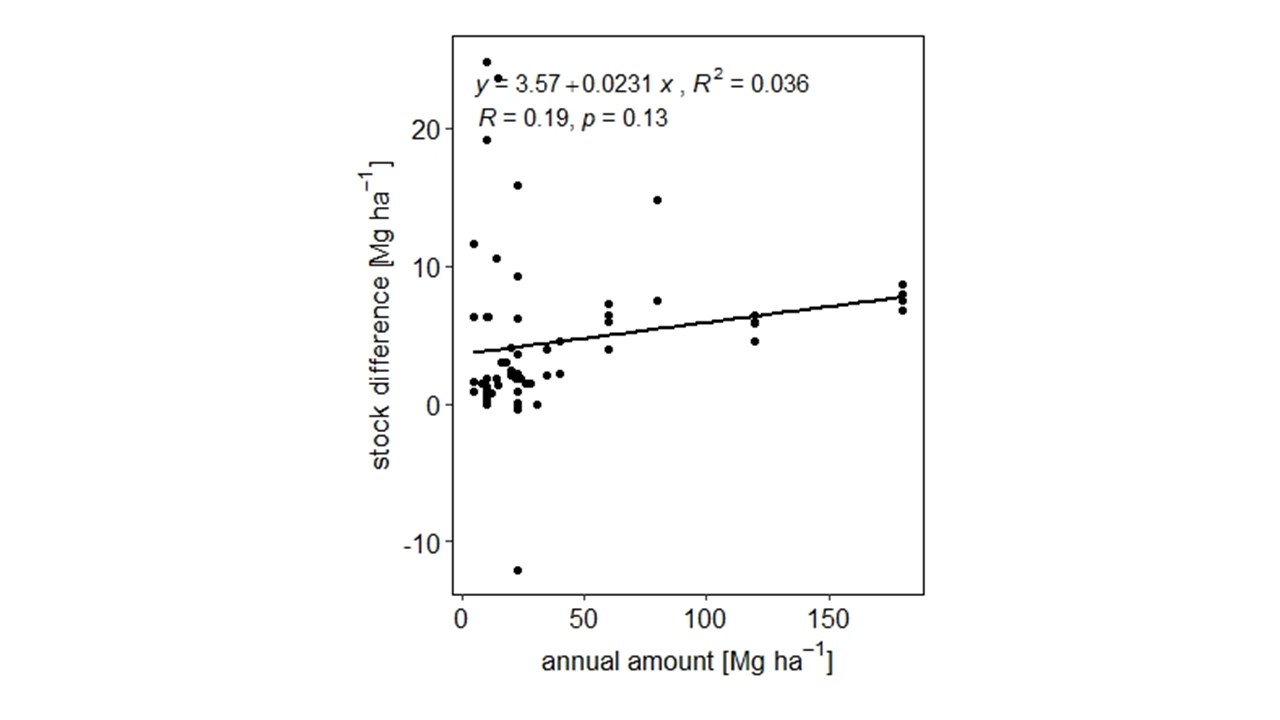
**

**m)**

**
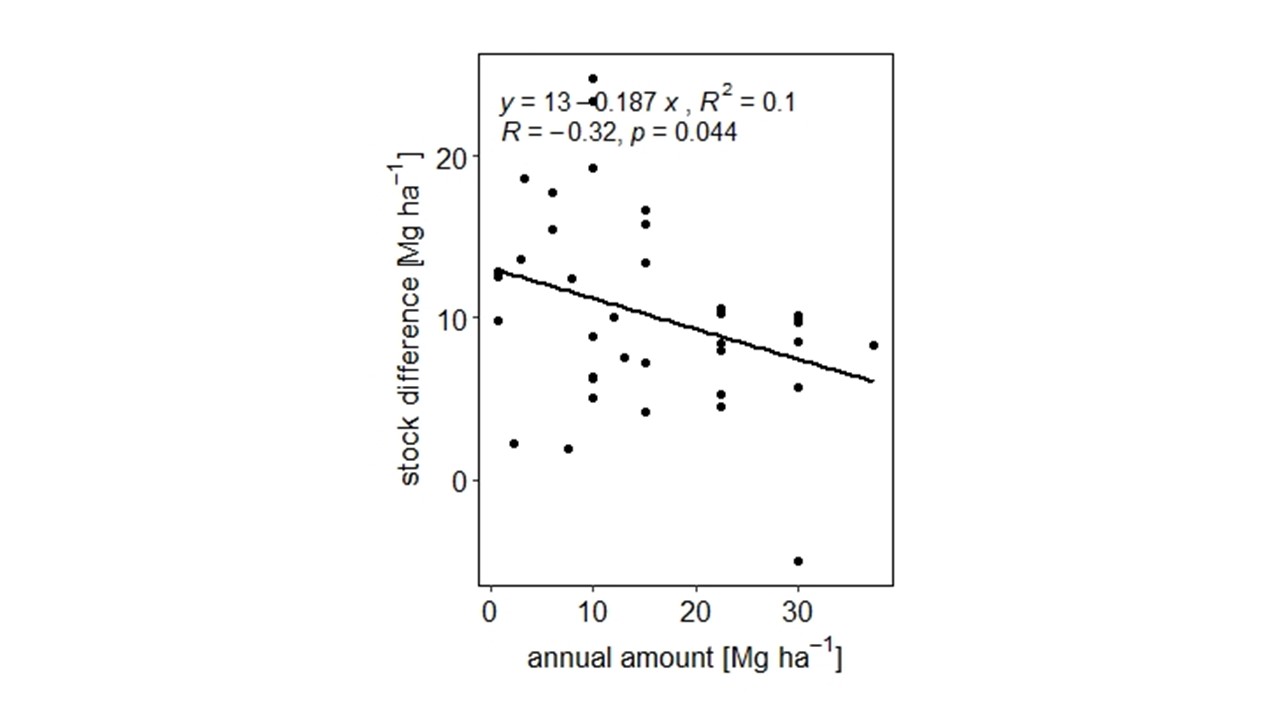
**

**n)**

**
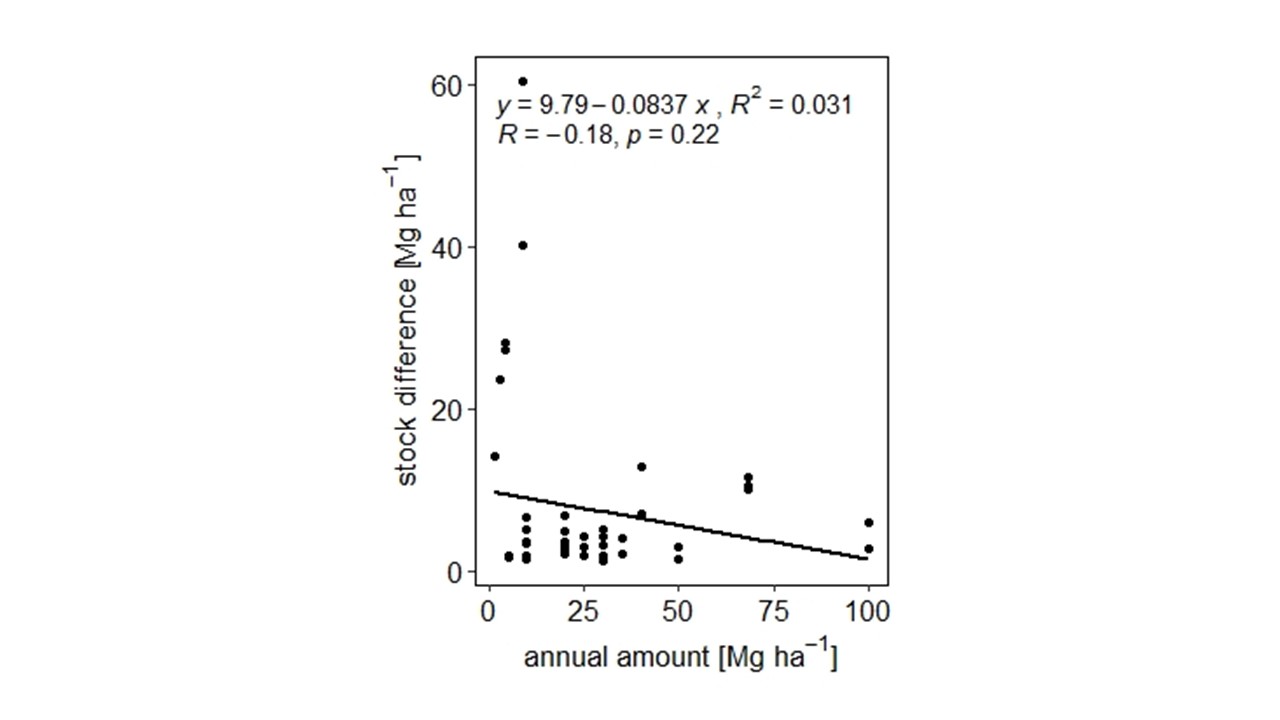
**

**o)**

**
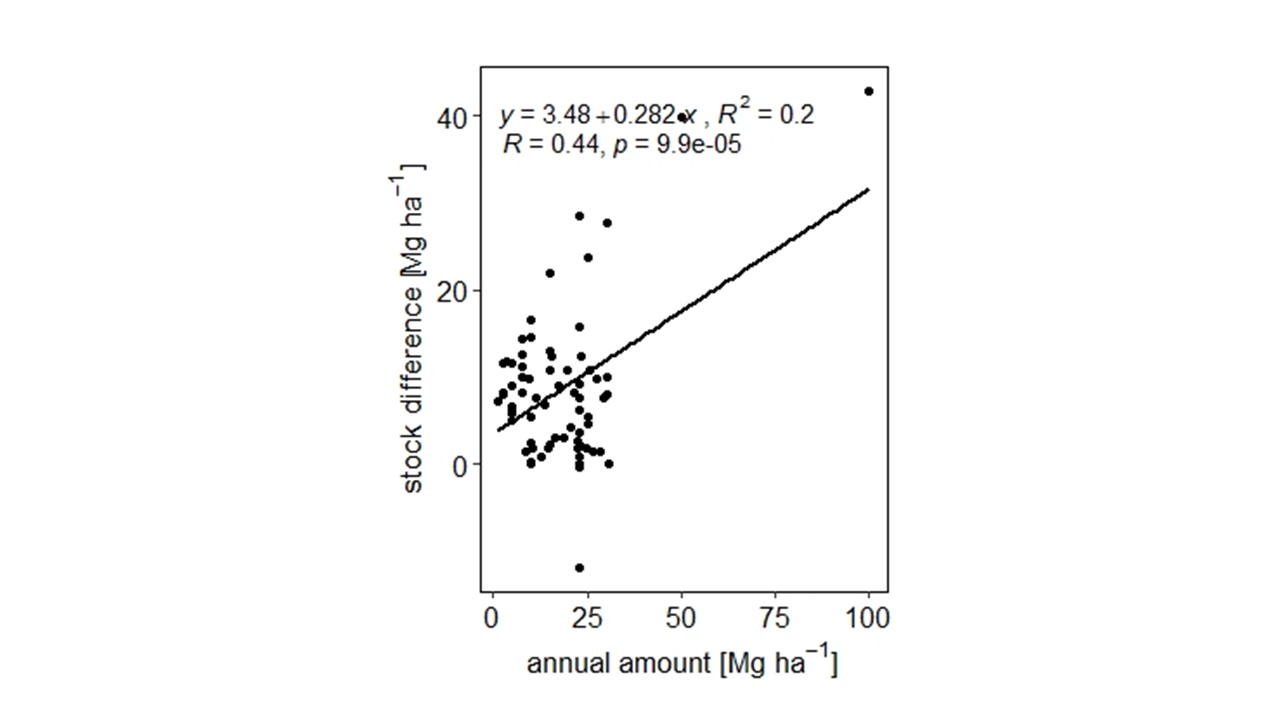
**

**p)**

**
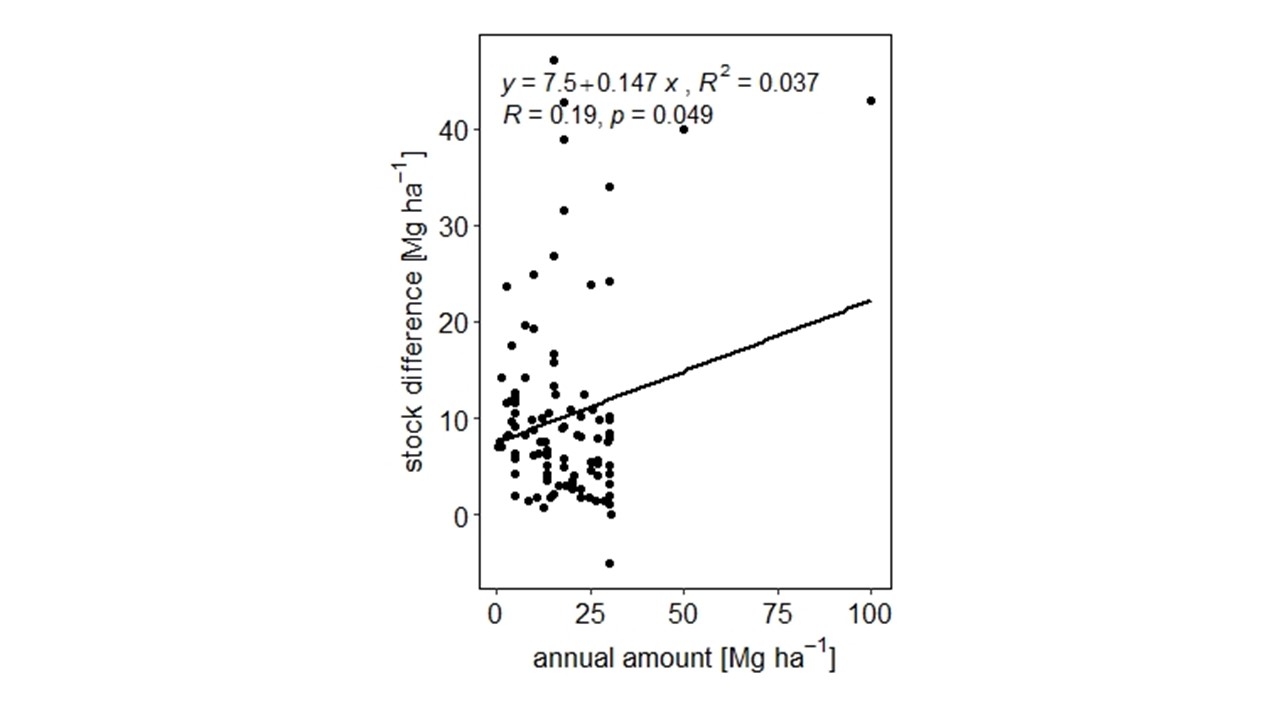
**

**q)**

**
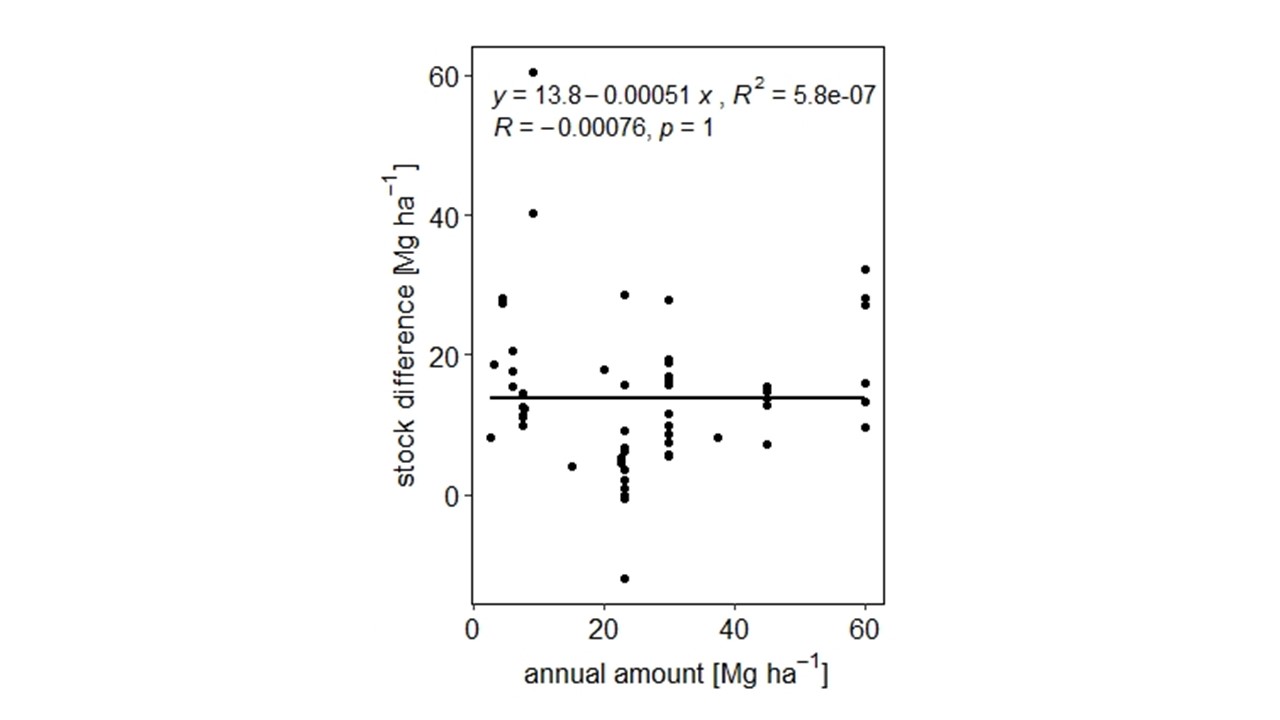
**

**r)**

**
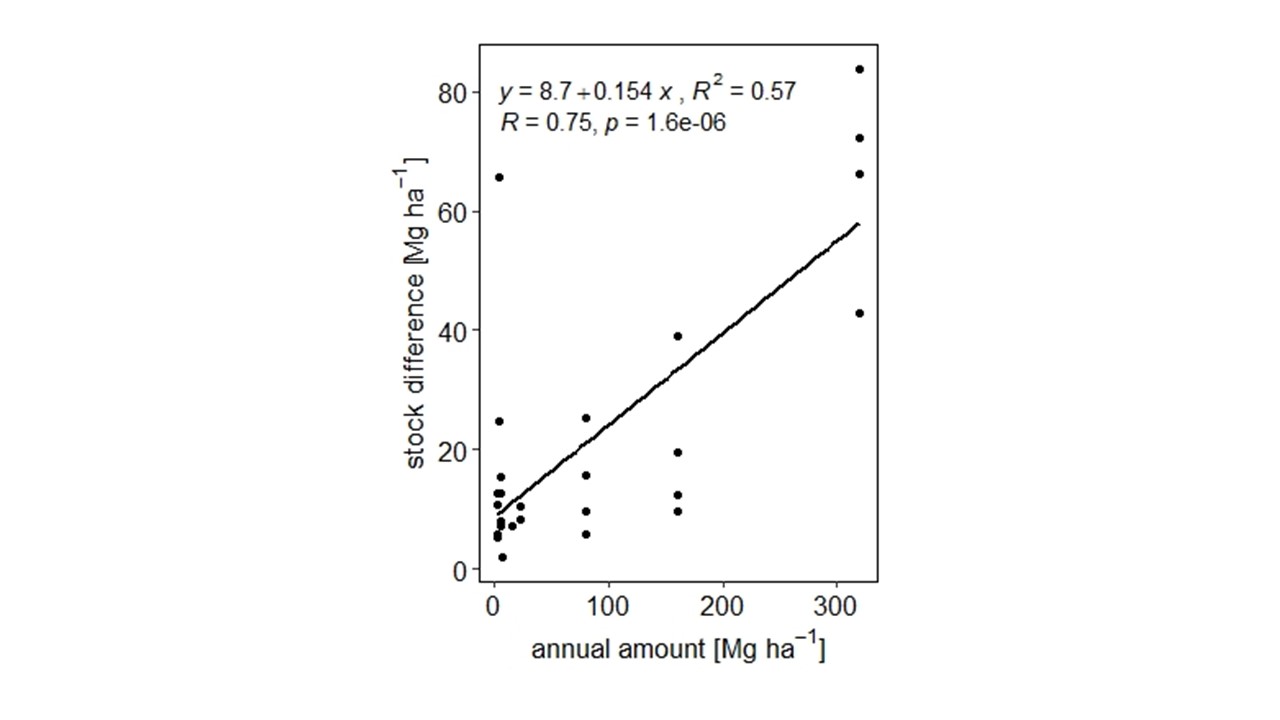
**

**s)**

**
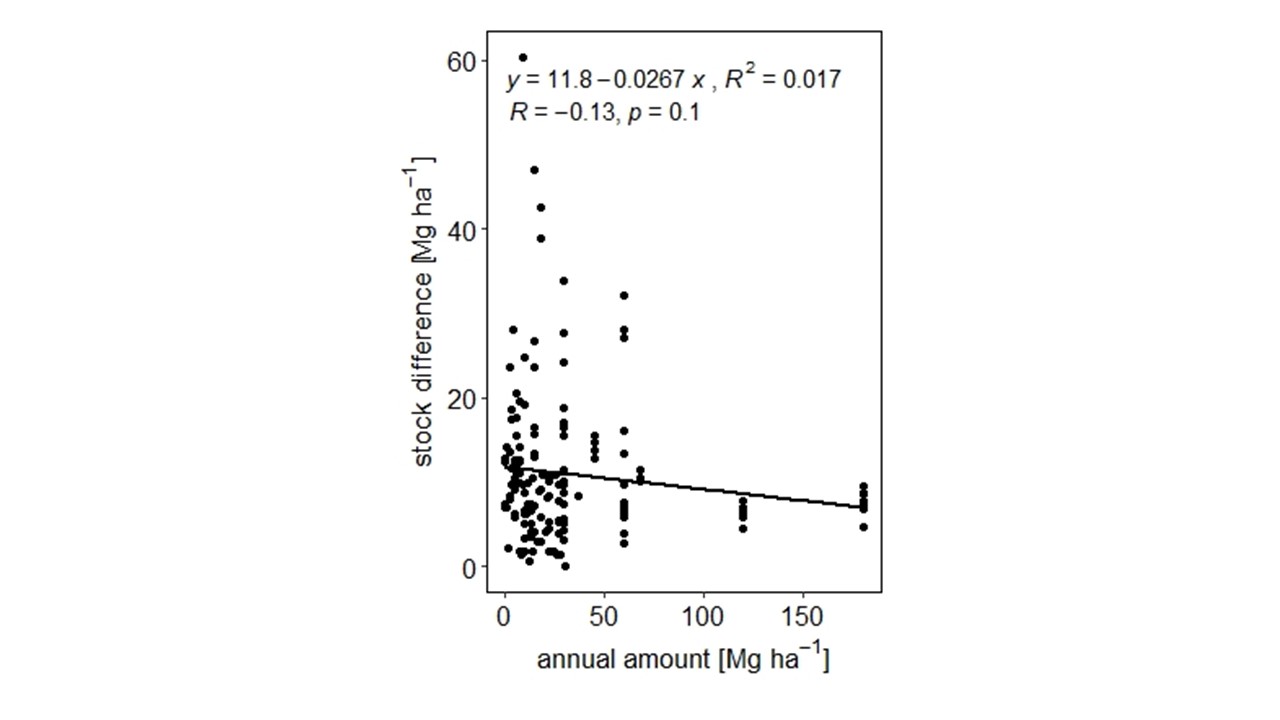
**

**t)**

**
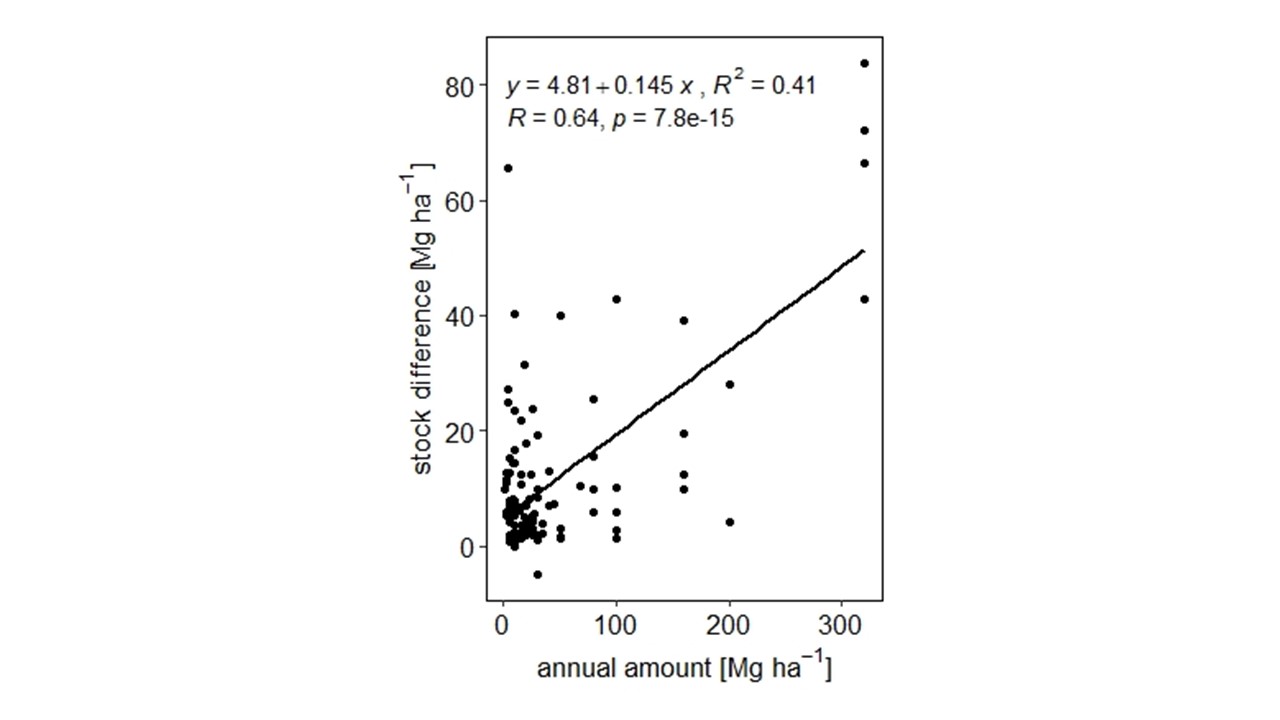
**

**Figure S15.** Relationship between SOC stock difference (Mg ha^-1^) and the annual manure input (Mg ha^-1^) under reduced tillage (a) and conventional tillage (b), the annual manure input in clay soils (c), silt soils (d), loam soils (e) and sand soils (f), the annual manure input under non-tropical (g) and sub-tropical (h) climate conditions, the annual manure input in sampling depths <=15 cm (i), 16-20 cm (j), 21-30 cm (k) and >30 cm (l), the annual manure input in acidic soils (m), alcalic (n) and pH neutral soils (o), the annual manure input in soils with low initial SOC (p), intermediate initial SOC (q) and high initial SOC (r) and the annual manure input in treatments with additional added chemical fertilizer (NPK) (s) and without additional NPK fertilizer (t). R^2^ represents the coefficient of determination.
